# Targeting the Medial Prefrontal Cortex with Transcranial Focused Ultrasound Alleviates Adolescent Sleep-Deprivation-Induced Persistent Postsurgical Pain in Adulthood

**DOI:** 10.34133/research.1437

**Published:** 2026-09-23

**Authors:** Jing Yang, Han Bo, Pin Lv, Tiantian Sun, Qingling Duan, Huijie Zhu, Wei Zhang, Xiaoping Gu, Hairong Zheng, Bing Zhang, Zhengliang Ma

**Affiliations:** ^1^ Department of Anesthesiology, Nanjing Drum Tower Hospital, Affiliated Hospital of Medical School, Nanjing University, Nanjing 210008, China.; ^2^ Department of Radiology, Nanjing Drum Tower Hospital, Affiliated Hospital of Medical School, Nanjing University, Nanjing 210008, China.; ^3^ Medical School, Nanjing University, Nanjing 210008, China.

## Abstract

Adolescent sleep deprivation (SD) increases latent vulnerability to pain, a major risk factor for persistent postsurgical pain (PPSP) in adulthood, yet the underlying mechanisms remain unclear. Given the limitations of current pharmacological treatments, noninvasive transcranial focused ultrasound (TFUS) offers a promising neuromodulatory alternative; however, its therapeutic efficacy for PPSP remains unknown. Here, we utilized a mouse model of adolescent SD followed by an adult plantar incision to elucidate how early sleep loss drives long-term PPSP and to systematically investigate the therapeutic potential and underlying mechanisms of TFUS. By integrating resting-state functional magnetic resonance imaging, chemogenetics, and behavioral assays, we demonstrated that medial prefrontal cortex glutamatergic (mPFC^Glu^) hypoactivity mediates this SD-driven PPSP development. To evaluate TFUS intervention, we first screened various stimulation paradigms and identified that 200-Hz pulse repetition frequency TFUS optimally activated mPFC^Glu^ neurons and effectively alleviated PPSP. Mechanistically, guided by single-nucleus RNA sequencing, we found that TFUS activates the mechanosensitive transient receptor potential channel 5 (TRPC5) to promote Homer1-dependent glutamatergic synaptic plasticity. This molecular cascade subsequently ameliorates mPFC^Glu^ hypoactivity and normalizes aberrant network-level gamma oscillations and spike–gamma phase locking. Collectively, our findings suggest that adolescent SD precipitates adult PPSP via mPFC^Glu^ dysfunction and that TFUS alleviates this condition through an integrated pathway linking molecular mechanosensation to network rhythm recovery. These results offer a novel therapeutic paradigm and provide a mechanistic foundation for developing noninvasive TFUS strategies to manage PPSP in vulnerable patients.

## Introduction

Adolescent sleep deficiency represents a major public health concern with lasting physiological and psychological consequences [1,2]. Driven by rising academic and social pressures, this deficiency has become increasingly prevalent during adolescence—a critical window for brain maturation. Recent studies show that sleep deficiency affects over 50% of the adolescent population and is linked to broader neurobehavioral sequelae in adulthood, including long-term memory impairment [3], asocial deficits [4], and, notably, enhanced pain sensitivity [5–8]. Throughout life, individuals inevitably encounter various physical traumas, such as accidental injuries or surgical procedures. Consequently, there is growing interest in whether a history of adolescent sleep deficiency predisposes individuals to more severe outcomes following such events. Because heightened vulnerability to pain is a major risk factor for the development of persistent postsurgical pain (PPSP), it is highly plausible that early sleep deficiency impairs long-term postoperative recovery. However, the precise causal link between adolescent sleep deprivation (SD) and adult PPSP remains unexplored. To address this, we developed a mouse model of adolescent SD followed by adult plantar incision to assess whether this early sleep deficiency drives central maladaptation and the subsequent development of PPSP.

PPSP affects up to 50% of patients and imposes a multidimensional burden [9,10], reducing the quality of life and increasing the risks of physical and mental comorbidities [11,12]. Furthermore, current pharmacological treatments for PPSP are often limited by slow onset, short duration, and frequent adverse effects. Although opioids remain central to postoperative pain management, their association with rising overdose mortality and side effects such as nausea and vomiting has precipitated a public health crisis [13–15]. This highlights the urgent need for innovative, nonpharmacological interventions to avoid opioid-related risks (abuse, overdose, and addiction) [13,16] and address comorbidities. Consequently, targeted neuromodulation strategies have gained prominence [17,18].

Among neuromodulation technologies, transcranial focused ultrasound (TFUS) stands out for its noninvasive, millimeter-precise energy delivery without ionizing radiation [19,20]. Combined with its safety, portability, and cost-effectiveness, TFUS has emerged as a powerful tool enabling region-specific neuromodulation with neuroprotective potential [21,22] and has been applied in various central nervous system disorders, including Alzheimer’s disease, Parkinson’s disease, and pain [23,24]. Both preclinical and clinical studies demonstrate that TFUS can alleviate pain by targeting specific neural regions. For example, In et al. [25] showed that TFUS targeting the posterior insula reduces the temporal summation of pain, while dorsal anterior cingulate cortex stimulation attenuates acute heat pain and autonomic responses [26]. Similarly, Kim et al. [27] found that repeated TFUS to the somatosensory cortex or insula in a sickle cell pain model provided prolonged analgesia and normalized aberrant theta and beta oscillations. Additionally, Phan et al. [28] revealed that theta–gamma patterned ultrasound persistently alleviated neuropathic pain by activating astrocytic transient receptor potential A1 (TRPA1) channels, thereby restoring the excitation/inhibition balance in the spinal dorsal horn. Although stimulation parameters and target regions varied across these studies, they collectively substantiate the immense potential of TFUS in pain management. Crucially, these findings indicate that the therapeutic mechanisms of TFUS primarily involve the modulation of neural oscillations and the activation of mechanosensitive ion channels [29]. While numerous studies have highlighted the critical role of neural oscillations in pain processing [30], few pharmacological agents can directly modulate them, making the oscillation-regulating capability of TFUS a highly promising avenue for pain therapy. However, it remains to be determined whether TFUS treatment—and specifically, which stimulation parameters—can effectively ameliorate PPSP. Moreover, whether TFUS exerts its therapeutic effects in PPSP by modulating neural oscillations or activating specific mechanosensitive ion channels is still unknown.

To address these gaps, the present study revealed how adolescent SD drives adult PPSP development and systematically investigated the therapeutic efficacy and underlying mechanisms of TFUS in this PPSP model. By integrating behavioral assays, fiber photometry, and functional magnetic resonance imaging (fMRI), our results validate the medial prefrontal cortex (mPFC) as the critical brain region mediating SD-induced PPSP, as well as a viable target for TFUS intervention. Furthermore, we demonstrate that TFUS ameliorates this pathology through an integrated mechanism: activation of the mechanosensor transient receptor potential (TRP) channel 5 (TRPC5) promotes synaptic plasticity, which subsequently restores cellular excitability and normalizes local network rhythms. This work provides a foundation for moving TFUS toward translational use in the clinical management of PPSP.

## Results

### Adolescent SD induces the development of PPSP in adulthood

To disrupt adolescent sleep, we employed an automated SD system that prevented mice from sleeping during the early light phase for 20 h per day (5 consecutive days within a mid-adolescent period [postnatal days 30 to 35, P30 to P35]; Fig. S1A and B). This protocol effectively suppressed rapid eye movement (REM) sleep and non-rapid eye movement (NREM) sleep and increased wakefulness (Fig. S1C, D, and G to I), as shown by a significant reduction in total REM sleep time and total NREM sleep time (Fig. S1E to I). Concurrently, we observed a significant increase in power spectral density (PSD) during wakefulness and NREM, accompanied by a marked decrease in PSD during REM following SD (Fig. S1J to L).

**Fig. 1.**
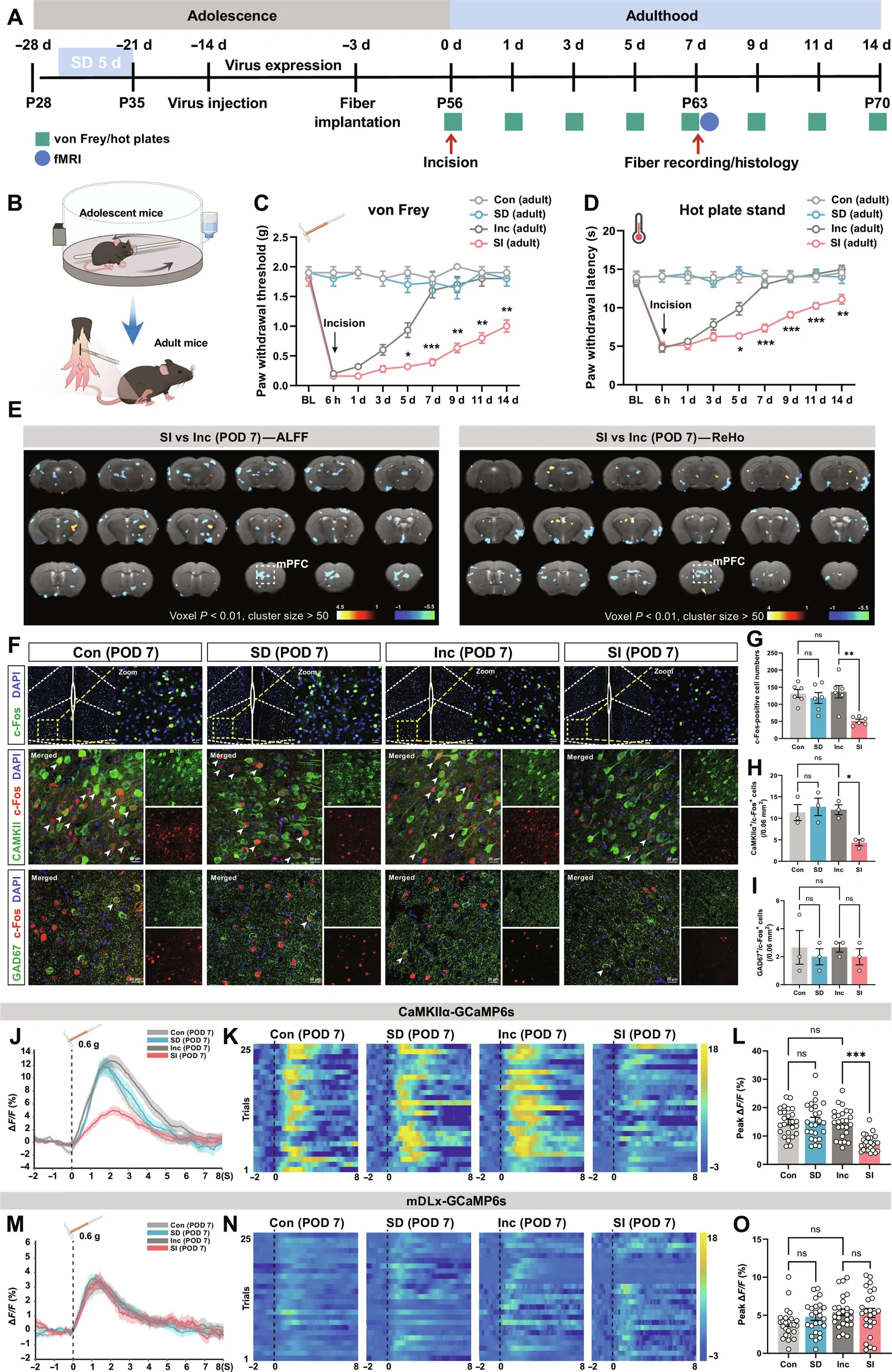
Adolescent SD induces PPSP in adult mice and alters mPFC^Glu^ neuronal activity. (A) Timeline of experimental procedures. (B) Schematic of the adolescent SD and adult incision model. (C and D) Time course assessment of nociceptive thresholds in the right hindpaw by the von Frey test and hot plate tests (*n* = 6; 2-way repeated-measures analysis of variance [ANOVA] with post hoc comparison). (E) Voxel-wise analysis of ALFF and ReHo changes showing the changed brain regions between the Inc and SI groups on POD 7 (*n* = 10 per group). (F to I) Representative images and quantification of overall c-Fos (*n* = 6 mice per group; one-way ANOVA), and c-Fos/CaMKIIα and c-Fos/GAD67 double immunofluorescence staining in the mPFC across the groups (*n* = 3 mice per group; one-way ANOVA). Scale bar, 20 μm. (J and K) Heatmap and average Ca^2+^ transients of mPFC excitatory neurons evoked by von Frey stimulation (*n* = 5 mice per group, with 5 trials conducted per mouse). (L) Statistics of peak amplitudes (one-way ANOVA) for fluorescent Ca^2+^ signals in mPFC excitatory neurons. (M and N) Heatmap and average Ca^2+^ transients of mPFC inhibitory neurons evoked by von Frey stimulation (*n* = 5 mice per group, with 5 trials conducted per mouse). (O) Statistics of peak amplitudes (one-way ANOVA) for fluorescent Ca^2+^ signals in mPFC inhibitory neurons. PPSP, persistent postsurgical pain; BL, baseline; mPFC, medial prefrontal cortex; mPFC^Glu^, mPFC glutamatergic‌; Con, control; SD, sleep deprivation; Inc, incision; SI, sleep deprivation combined with incision; POD 7, postoperative day 7; ALFF, amplitude of low-frequency fluctuation; ReHo, regional homogeneity. Data are presented as mean ± standard error of the mean (SEM). SI vs Inc, *P < 0.05; **P < 0.01; ***P < 0.001.

Control littermates (Con) were placed in the same SD chamber without activation. Both Con and SD mice were then raised under standard conditions until adulthood (8 weeks of age, P56, Fig. 1A). At this young-adult stage, all mice exhibited normal baseline pain thresholds (P56, Fig. S2A to C) and sleep architecture (Fig. S2D to M). Mechanical allodynia and thermal hyperalgesia were assessed using the paw withdrawal threshold (PWT) and paw withdrawal latency (PWL), respectively. However, following a right hindpaw incision surgery at P56 (Fig. 1B), Con mice (incision [Inc] group) recovered normal pain thresholds within 7 d. In contrast, mice that had undergone adolescent SD (SD + incision [SI] group) showed persistent pain lasting until postoperative day (POD) 14 (Fig. 1C and D), indicating delayed recovery and the development of PPSP. To determine if this behavioral phenotype is consistent across sexes, we performed the same SD and incision procedures in female mice. Similar to males, female mice subjected to adolescent SD exhibited prolonged postoperative hypersensitivity (Fig. S3A to C). Collectively, these data indicate that while adolescent SD did not alter the basal pain threshold in adulthood, it significantly exacerbated postoperative hyperalgesia and delayed pain resolution following plantar incision in both sexes, ultimately driving the transition to PPSP. Due to the weight constraints of the head-mounted apparatus for in vivo recordings, subsequent mechanistic experiments focused on male mice.

### mPFC glutamatergic neuronal hypoactivity mediates SD-driven PPSP development

The above results suggest that adolescent SD induces a state of latent vulnerability in the central nervous system, rather than directly altering peripheral nociception. To identify the specific brain regions mediating this central maladaptation, we performed resting-state fMRI comparing the Inc and SI groups (Fig. 1A) on POD 7 (P63). We selected this specific time point for all subsequent mechanistic investigations because it marked the maximal divergence in pain thresholds between groups, representing a critical window for the transition from acute to persistent pain. Voxel-wise amplitude of low-frequency fluctuation (ALFF) and regional homogeneity (ReHo) analyses both revealed significant reductions across multiple brain regions in the SI group (Fig. 1E). (A full tabulation of these regions is provided in Table S2.) Among the regions exhibiting consistent reductions in both ALFF and ReHo, we focused on the mPFC. The mPFC is a well-established node in the descending pain inhibition system and a common target for neuromodulatory interventions. Consequently, we selected the mPFC for further validation and targeted intervention.

Consistently, c-Fos staining demonstrated that the Con, SD, and Inc groups exhibited comparable mPFC neuronal activities on POD 7, but the SI group showed a significant reduction compared to the Inc group (Fig. 1F and G). To determine the specific cell types involved, double immunofluorescence staining was performed. The density of c-Fos^+^/CaMKIIα^+^ neurons was significantly decreased in the SI group compared to that in the Inc group, whereas the density of c-Fos^+^/GAD67^+^ neurons showed no significant differences across all groups (Fig. 1F to I). In line with these histological findings, fiber photometry was performed using rAAV-CaMKIIα-GCaMP6s (excitatory) and rAAV-mDLx-GCaMP6s (inhibitory) (Fig. 1H). Upon mechanical stimulation, calcium signals in mPFC glutamatergic‌ (mPFC^Glu^) neurons were similar among the Con, SD, and Inc groups, whereas the SI group showed significantly lower signals compared to the Inc group (Fig. 1J to L). Inhibitory signals remained unchanged across all 4 groups (Fig. 1M to O). Collectively, these findings demonstrate that adolescent SD creates a latent vulnerability that manifests as specific hypoactivity of mPFC^Glu^ only upon surgical incision, thereby underlying PPSP and serving as a functional link mediating the long-term effects of adolescent SD into adulthood.

The above correlative results prompted us to explore the potential causal roles of mPFC^Glu^ in regulating postoperative pain. To investigate this hypothesis, we bilaterally injected AAV-CaMKIIα-hM3Dq-mCherry and AAV-CaMKIIα-mCherry into the mPFC of SI mice (SI-hM3Dq mice and SI-mCherry mice; Fig. S4A and B) and similarly injected AAV-CaMKIIα-hM4Di-mCherry or control vectors into the mPFC of Inc mice (Inc-hM4Di and Inc-mCherry mice; Fig. S4C). Following once-daily intraperitoneal injections of clozapine *N*-oxide for 7 consecutive days (PODs 1 to 7), chemogenetic activation of mPFC^Glu^ successfully restored both mechanical and thermal pain thresholds to baseline levels by POD 7 in hM3Dq mice, unlike mCherry controls (Fig. S4C and D). Conversely, chemogenetic inhibition of mPFC^Glu^ neurons significantly delayed postoperative pain recovery, reproducing a PPSP-like phenotype compared to mCherry controls (Fig. S4F and G). Taken together, specific mPFC^Glu^ activation yields analgesia in SI mice, while its inhibition induces PPSP-like behaviors in normal Inc mice, confirming that mPFC^Glu^ hypoactivity drives the presentation of PPSP.

### Characterizing TFUS stimulation and selecting the optimal PRF for targeting mPFC^Glu^ neurons

Considering the aforementioned hypoactivity of mPFC^Glu^ neurons under pathological states, developing feasible strategies to activate mPFC^Glu^ may have great translational potential for treating PPSP. Both clinical practice and preclinical studies have documented the efficacy of TFUS in pain management. However, substantial limitations still remain, i.e., inconsistent targeting of brain regions and a lack of standardized treatment protocols, which might be attributed to unclear mechanisms. To address this, we took TFUS as a strategy for the modulation of mPFC^Glu^ neuronal activity and explored how it operates at the network and cellular levels.

**Fig. 2.**
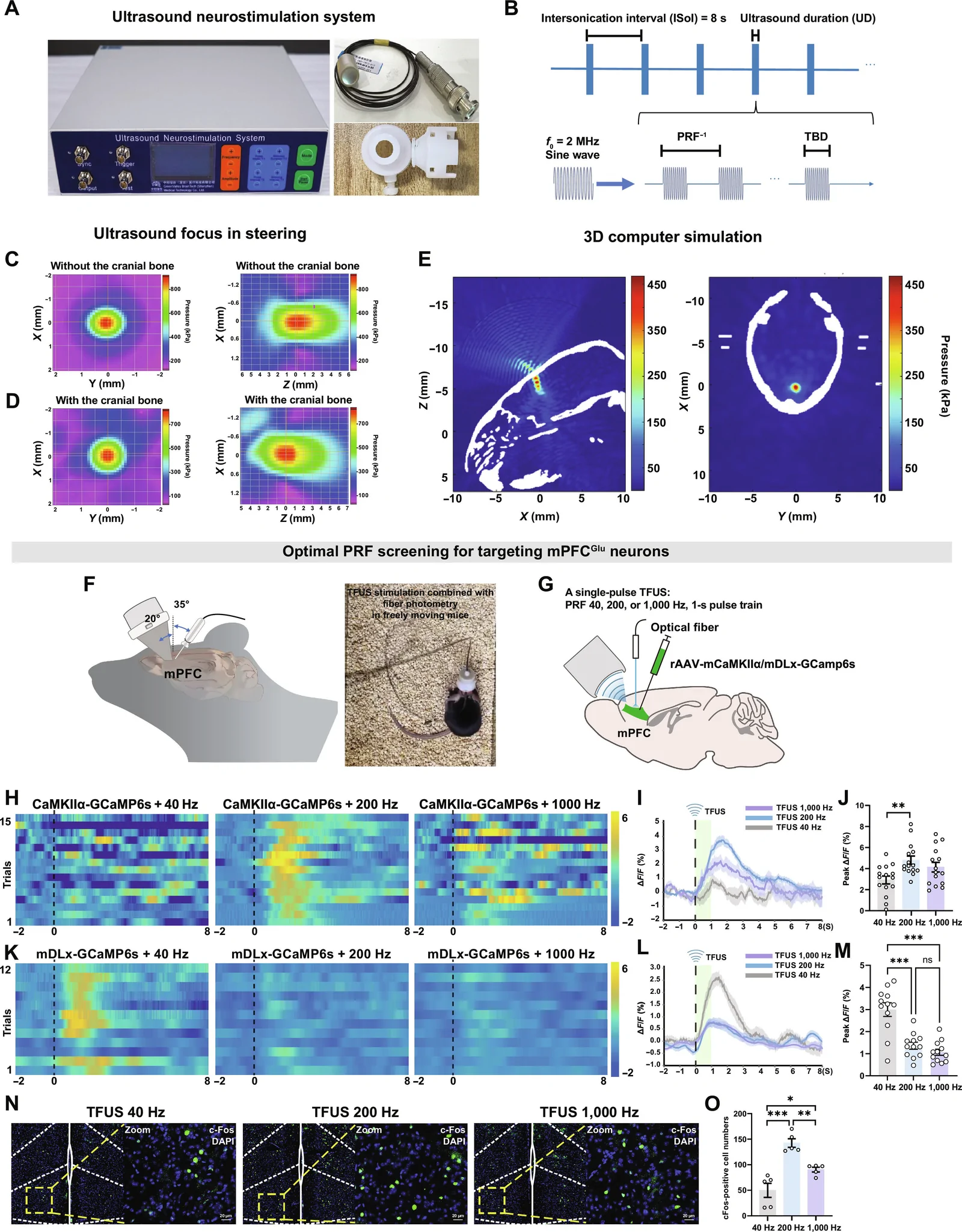
Optimization of TFUS parameters: pressure field characterization and PRF selection for mPFC^Glu^ targeting. (A) Photographs of the ultrasound device, transducer, and collimator. (B) An illustration of the ultrasound temporal profile used. (C and D) One transverse (*X*–*Y* plane) and one sagittal (*X*–*Z* plane) scan of ultrasound pressure distribution with and without the cranial bone using a hydrophone-based US field mapping system. (E) 3-dimensional (3D) simulated maximum acoustic pressure distribution in the sagittal (*X*–*Z*) and transverse (*X*–*Y*) planes within the mouse skull (white), indicating that the expected acoustic focus aligns with and covers the intended mPFC target region. (F) A custom collimator guiding focused ultrasound to the hair-removed scalp of a free-moving mouse model with an incidence angle of 20° and a fiber inserted through an integrated slot at an incidence angle of 35° into the right mPFC. These angles were calculated to allow the acoustic focus and the fiber tip to converge at the target mPFC coordinates (AP +1.9, ML +0.35, DV −1.9). (G) Schematic presentation of the viral injection and TFUS stimulation protocol. (H and I) Heatmap and average Ca^2+^ transients of mPFC^Glu^ across different ultrasound PRFs (*n* = 5 mice per group, with 3 trials conducted per mouse). (J) Statistical comparison of peak amplitudes (one-way analysis of variance [ANOVA]). (K and L) Heatmap and average Ca^2+^ transients of mPFC^GABA^ across different ultrasound PRFs (*n* = 4 mice per group, with 3 trials conducted per mouse). (M) Statistical comparison of peak amplitudes (one-way ANOVA). (N and O) Representative images and quantification of c-Fos immunohistochemistry (*n* = 5 mice per group; one-way ANOVA). Scale bar, 20 μm. mPFC, medial prefrontal cortex; mPFC^Glu^, mPFC glutamatergic‌; TFUS, transcranial focused ultrasound; *f*_0_, fundamental frequency; PRF, pulse repetition frequency; DC, duty cycle; TBD, tone-burst duration; UD, ultrasound duration; ISoI, intersonication interval; AP, anteroposterior; ML, mediolateral; DV, dorsoventral. Data are presented as mean ± standard error of the mean (SEM). *P < 0.05; **P < 0.01; ***P < 0.001.

The in vivo experimental setup and ultrasound waveforms are shown in Fig. 2A and B. TFUS was targeted over the right mPFC, using tone-burst sinusoidal waves with a fixed frequency (2 MHz), a fixed duty cycle (DC, 10%), and varied pulse repetition frequencies (PRFs) at 40, 200, or 1,000 Hz (Table S1). Each trial included an 8-s intersonication interval (ISoI). Ex vivo hydrophone mapping evaluated the spatial profile behind a freshly excised skull. Pressure distributions were mapped in the *X*–*Y* and *X*–*Z* planes with (Fig. 2D) and without the skull (Fig. 2C). Subsequent 3-dimensional (3D) reconstructions in sagittal and transverse planes (Fig. 2E) revealed that mechanical energy penetrated to a 4-mm depth, with the spatial-peak pressure concentrated within 2 mm behind the skull.

The frequency parameter, specifically the PRF, is considered a main determinant of TFUS efficacy in both clinical trials and animal studies [31–33], potentially underlying its neuron-type-specific functional effects. To identify the optimal PRF for mPFC^Glu^ activation, we systematically tested 40-, 200-, and 1,000-Hz PRFs, combining TFUS with fiber photometry in freely moving mice (Fig. 2F). A custom collimator facilitated the precise oblique placement of an optical fiber just above the mPFC following the injection of AAV-CaMKII-GCaMP6s or AAV-mDlx-GCaMP6s (Fig. 2G). Delivering TFUS at the 3 PRFs (10% DC, 1-s pulse train) revealed that both the 200- and 1,000-Hz protocols significantly increased excitatory neuronal calcium signals during stimulation (0 to 8 s post-onset), while 40 Hz produced minimal changes (Fig. 2H to J). Conversely, inhibitory neuron recordings showed that 40-Hz PRF TFUS increased calcium signals, whereas the 200- and 1,000-Hz PRF protocols elicited minimal activation (Fig. 2K to M). We further confirmed this mPFC-specific activation using c-Fos immunohistochemistry. Aligning with the excitatory photometry results, overall c-Fos^+^ neuron counts were highest at 200-Hz PRF, intermediate at 1,000-Hz PRF, and lowest at 40-Hz PRF (Fig. 2N and O). This activation was strictly localized to the mPFC, showing no significant alterations in adjacent regions (Fig. S5A to D) or the auditory cortex (Fig. S5E and F). These findings demonstrate that the 200-Hz PRF TFUS protocol induces robust, spatially precise excitatory neuronal activation in the mPFC without collateral auditory confounds.

### TFUS ameliorates adolescent SD-induced PPSP in adulthood

Given that both the 200- and 1,000-Hz PRF TFUS protocols triggered robust neuromodulation, we next tested their therapeutic efficacy. A single TFUS session was delivered to the mPFC of SI mice (Fig. S6A and B). The parameters were as follows: 20-min duration, 10% DC, a spatial-peak temporal-average intensity (*I*_SPTA_) of 84.38 mW/cm^2^, and a spatial-peak pulse-average intensity (*I*_SPPA_) of 6.75 W/cm^2^. All applied acoustic parameters were maintained within the Food and Drug Administration (FDA) safety limits for diagnostic ultrasound imaging (*I*_SPTA_ < 720 mW/cm^2^ and *I*_SPPA_ < 190 W/cm^2^) [34]. On POD 7, a single session of either 200- or 1,000-Hz PRF TFUS significantly increased mechanical (PWT) and thermal (PWL) pain thresholds from baseline. However, this analgesic effect was transient, lasting only between 30 min and 2 h (Fig. S6C and D).

**Fig. 3.**
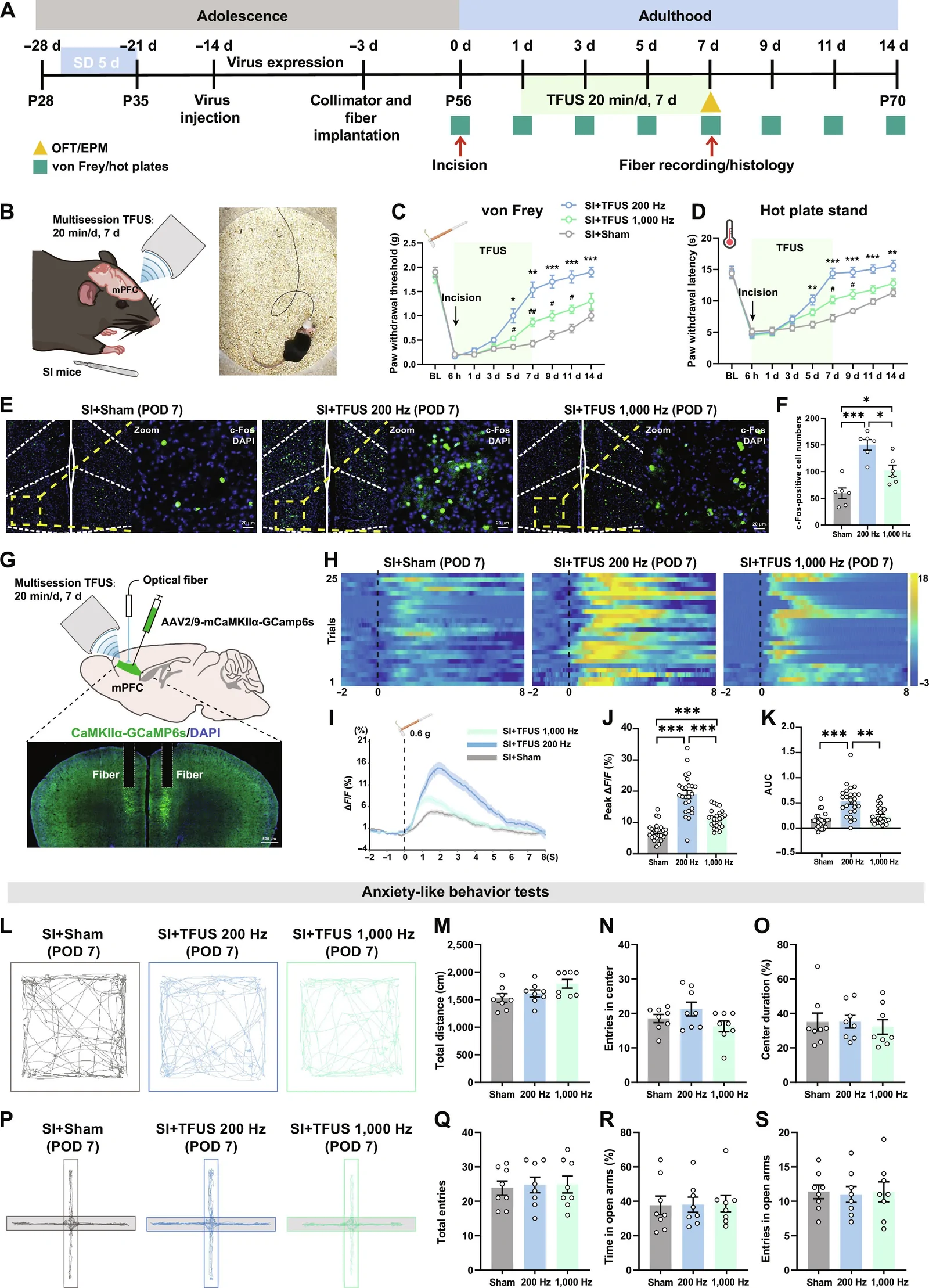
Multisession TFUS alleviates PPSP by restoring mPFC^Glu^ neuronal activity. (A and B) Similar to Fig. 1A but combined with multisession TFUS stimulation. On POD 7, behavioral testing and fiber photometry recordings were performed in separate cohorts of mice 3 h after the final TFUS or sham stimulation. Brain tissues for histological analysis were collected 1 h after the behavioral tests. (C and D) Effects of multisession TFUS at sham, PRF 200 Hz, or PRF 1,000 Hz on nociceptive thresholds in SI mice (*n* = 6; 2-way repeated-measures analysis of variance [ANOVA] with post hoc comparison; * indicates SI+TFUS 200 Hz vs SI+Sham group, and # indicates SI+TFUS 1,000 Hz vs SI+Sham group). (E and F) Representative c-Fos immunohistochemistry images in the mPFC with quantitative summary (*n* = 6; one-way ANOVA). Scale bar, 20 μm. (G) Schematic presentation of the viral injection, TFUS stimulation protocol, and representative images of rAAV-CaMKIIα-GCaMP6s expressions in the mPFC (scale bar, 500 μm). (H and I) Heatmaps and the representative average Δ*F*/*F* traces of mPFC^Glu^ signals (*n* = 5 mice per group, with 5 trials conducted per mouse). (J and K) Statistics of peak amplitudes (Welch’s ANOVA test) and AUC (Kruskal–Wallis test) between changes across mice. (L to S) Behavioral tests, including the OFT and EPM, showing that multisession TFUS with a PRF of 200 or 1,000 Hz does not induce anxiety-like behaviors in SI mice (*n* = 8; one-way ANOVA). BL, baseline; AUC, area under the curve; mPFC, medial prefrontal cortex; mPFC^Glu^, mPFC glutamatergic‌; Inc, incision; SI, sleep deprivation combined with incision; TFUS, transcranial focused ultrasound; PPSP, persistent postsurgical pain; POD 7, postoperative day 7; PRF, pulse repetition frequency; OFT, open-field test; EPM, elevated plus maze. Data are presented as mean ± standard error of the mean (SEM). *P < 0.05; **P < 0.01; ***P < 0.001; ^#^*P* < 0.05; ^##^*P* < 0.01.

To achieve sustained effects, multisession TFUS was administered for 7 d (20 min/d), with pain thresholds evaluated (Fig. 3A and B). On day 7, multisession TFUS, but not sham, restored pain thresholds to baseline, preventing persistent pain in SI mice (Fig. 3C and D). Notably, behavioral assays demonstrated that the analgesic efficacy of 200-Hz PRF TFUS was significantly superior to that of 1,000-Hz PRF. This frequency-dependent pattern was corroborated by the c-Fos immunohistochemistry results (Fig. 3E and F). Double staining further confirmed that TFUS treatment, particularly at 200-Hz PRF, significantly increased the density of c-Fos^+^/CaMKIIα^+^ neurons in SI mice, without altering the c-Fos^+^/GAD67^+^ population (Fig. S7A to C). Furthermore, fiber photometry revealed that mechanical stimulation of the paw with a 0.6-g von Frey filament (Fig. 3G) evoked a significantly higher excitatory neuronal calcium signal intensity in the SI+TFUS 200-Hz PRF and 1,000-Hz PRF groups compared to the SI+Sham group, with the most pronounced effect at 200-Hz PRF (Fig. 3H to K). Importantly, the open-field test (OFT) (Fig. 3L to O) and elevated plus maze (EPM) tests (Fig. 3P to S) confirmed no adverse effects on locomotor or anxiety-like behavior. These findings demonstrate that 7-d multisession TFUS (particularly at 200-Hz PRF) effectively reverses cellular mPFC^Glu^ hypoactivity and alleviates PPSP without inducing motor or anxiety-related abnormalities. Building on this cellular-level efficacy, we next sought to elucidate the underlying therapeutic mechanisms across network and molecular dimensions.

### TFUS restores local mPFC activity and enhances pain-related network connectivity

To determine whether the therapeutic effect of TFUS involves local mPFC normalization or broader network-level recovery, we performed additional resting-state fMRI following the 7-d TFUS treatment. Analysis of spontaneous brain activity showed that TFUS mitigated the ALFF deficits in the mPFC of SI mice (Fig. S8A), indicating local functional restoration. We further assessed broader network changes by conducting a functional connectivity (FC) analysis focused on 8 established pain-related regions (comprising 106 subregions, including the mPFC, ACC, insular cortex, hippocampus, amygdala, medial thalamus, periaqueductal gray [PAG], and locus coeruleus). The group-averaged FC matrices for the SI+Sham and SI+TFUS groups are presented in Fig. S8B and C. Network-based statistic analysis (Fig. S8D and E) and visualization of the top 10% strongest connections (Fig. S8F and G) revealed that TFUS treatment enhanced overall FC within this pain network. Notably, the connectivity between the mPFC and the PAG was significantly strengthened. These results indicate that targeted TFUS at the mPFC not only restores local activity but also facilitates functional recovery across the broader descending pain modulatory network.

### TFUS normalizes aberrant gamma oscillations and spike–gamma coupling

**Fig. 4.**
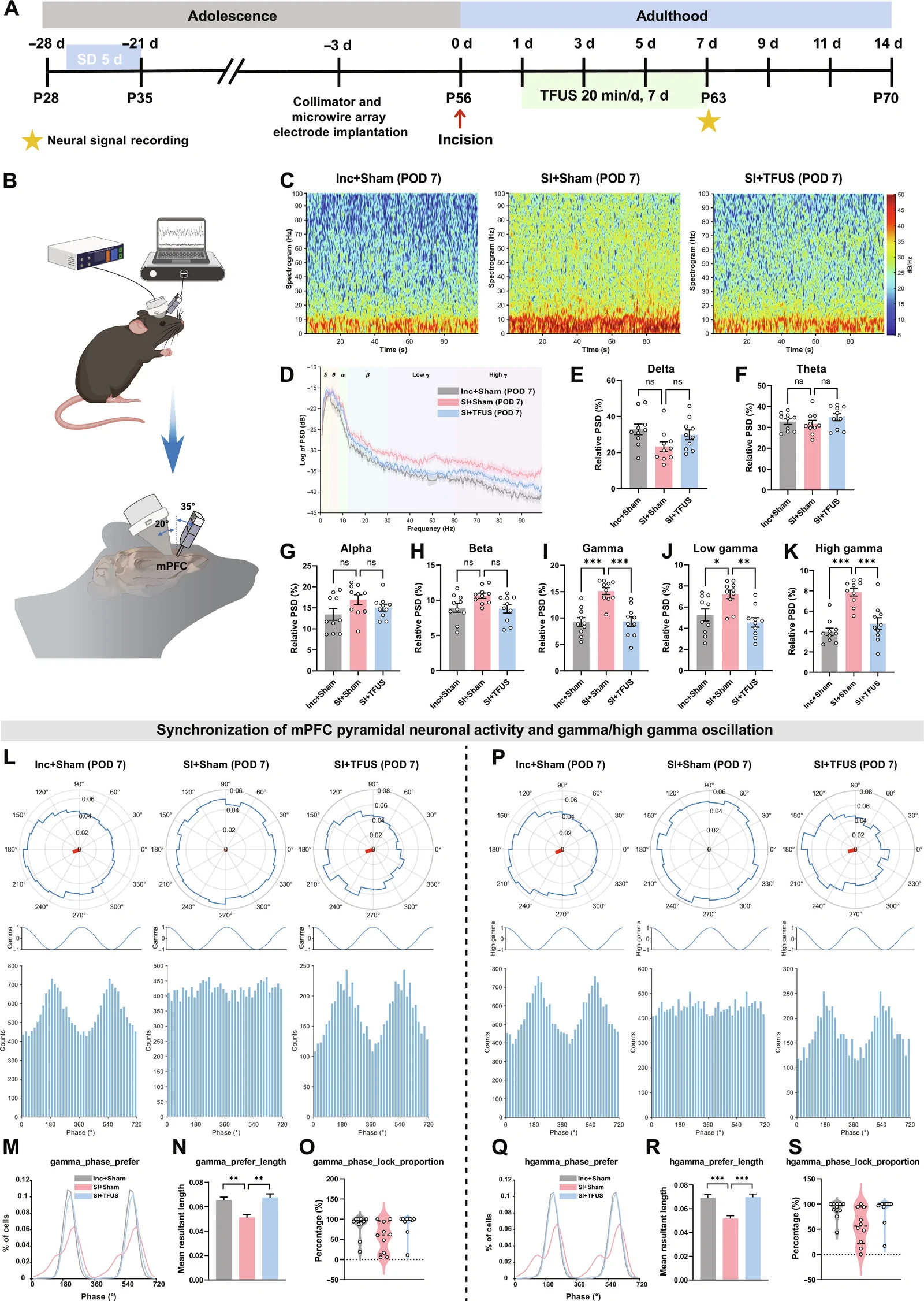
TFUS rescues mPFC rhythm abnormalities. (A) Similar to Fig. 1A but combined with multisession 200-Hz PRF TFUS stimulation and in vivo multichannel electrophysiology. On POD 7, in vivo electrophysiological recordings were performed 3 h after the final TFUS or sham stimulation. (B) As illustrated, the 16-channel electrode array is inserted into the mPFC via the cranial burr hole with an incidence angle of 35°. (C) A time–frequency representation of spectral power in the mPFC across the Inc+Sham, SI+Sham, and SI+TFUS groups (*n* = 10). (D to K) Comparison of relative PSD across frequency bands among the 3 groups (one-way analysis of variance [ANOVA]). (L) Representative phase-locking plots in the mPFC relative to local gamma oscillations. Top: Polar histograms with red vectors indicating the mean resultant direction (angle) and length (magnitude). Bottom: Distribution of spike-phase angles, showing preferred gamma phase (angle) and locking strength (radius) for phase-locked neurons. (M and Q) Gamma and high-gamma phase preference plot. (N) The strength of mPFC neuron phase locking to local gamma oscillations (Kruskal–Wallis test). (O and S) The proportion of phase-locking mPFC neurons. (P) Representative phase-locking plots in the mPFC relative to local high-gamma oscillations. (R) The strength of mPFC neuron phase locking to local high-gamma oscillations (Kruskal–Wallis test). mPFC, medial prefrontal cortex; Inc, incision; SI, sleep deprivation combined with incision; POD 7, postoperative day 7; TFUS, transcranial focused ultrasound; PRF, pulse repetition frequency; PSD, power spectral density. Data are presented as mean ± standard error of the mean (SEM). *P < 0.05; **P < 0.01; ***P < 0.001.

Given the restored FC between the mPFC and broader pain networks, we hypothesized that the 200-Hz PRF might modulate local neural oscillations, which may underlie these macroscopic changes. To investigate this local neural rhythm mechanism, we performed in vivo electrophysiological recordings in freely moving mice using a 16-channel microelectrode array implanted in the mPFC (Fig. 4A and B). First, the analysis of relative PSD revealed that SI+Sham mice exhibited increased overall gamma power (30 to 100 Hz)—including both the low- (30 to 60 Hz) and high-gamma (60 to 100 Hz) subbands—compared to Inc+Sham controls (Fig. 4C to K). Other frequency bands remained unaffected (Fig. 4E to H). Crucially, repeated TFUS over 7 d completely normalized this aberrant gamma power to control levels (Fig. 4I to K).

Next, given the central role of mPFC^Glu^ (pyramidal) neurons, we investigated their synchronization with local gamma oscillations (Fig. 4L and M). Analysis showed that, while the proportion of phase-locked neurons remained unchanged in the SI group (Fig. 4O), their phase-locking strength (mean resultant length) was significantly reduced (Fig. 4N). TFUS intervention effectively restored this synchronization strength (Fig. 4I). Further analysis demonstrated that this impairment and subsequent TFUS-mediated restoration were specific to the high-gamma band (Fig. 4P to S), whereas low-gamma synchronization remained unaltered (Fig. S9A to D).

Collectively, these findings demonstrate that PPSP is characterized by enhanced gamma power and impaired gamma–spike synchronization in the mPFC. TFUS successfully reverses both pathophysiological features, providing a network-level basis for its analgesic effects.

### TRPC5 facilitates the ultrasonic activation of mPFC^Glu^ neurons

**Fig. 5.**
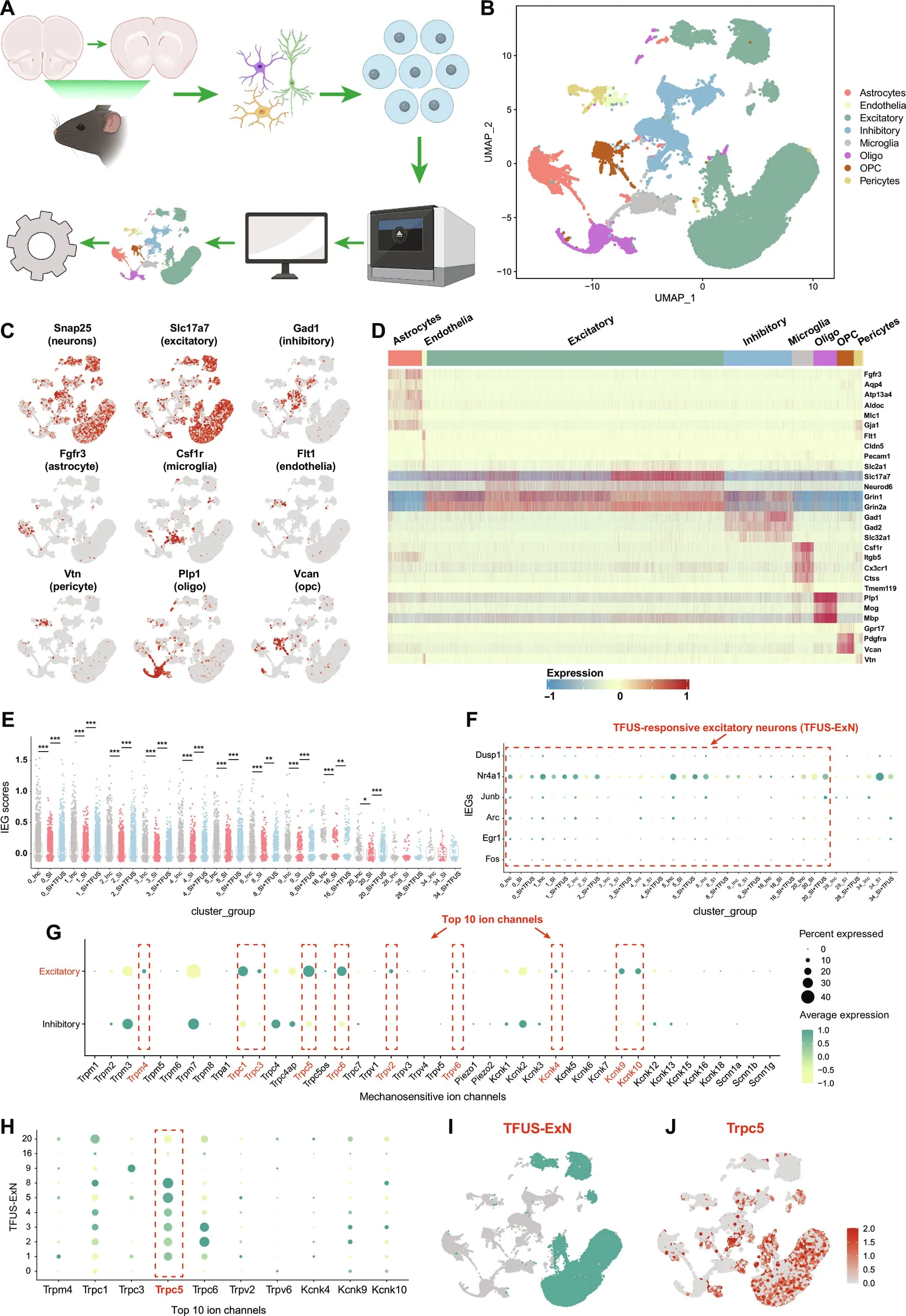
Molecular characterization of TFUS-responsive excitatory neurons (TFUS-ExN) in the mPFC. (A) Schematic of the experimental design. mPFC sections were isolated for single-nucleus RNA sequencing (snRNA-seq). Created with BioRender.com. (B) Uniform manifold approximation and projection (UMAP) plot showing the broad clustering of mPFC cell types based on the transcriptome. (C) Expression of cell-type-specific markers in each broad cell cluster color-highlighted on UMAP plots. (D) Single cell heatmap showing cell-type-specific gene marker expression in the different mPFC cell clusters. (E and F) Expression levels of IEGs in excitatory neurons across experimental groups (Inc+Sham, SI+Sham, and SI+TFUS). The TFUS-ExN clusters are highlighted by a red dashed box. (G) Expression profiles of TRP, PIEZO, KCNK, and SCNN channel genes across excitatory and inhibitory neurons. The red dashed boxes highlight the top 10 genes predominantly expressed in excitatory neuronal populations. (H) Expression of the top 10 mechanosensitive ion channels across all TFUS-ExN. (I) The TFUS-ExN are color coded (green) on the UMAP plot. (J) The expression level of TRPC5 is color coded (red) on the UMAP plot. mPFC, medial prefrontal cortex; Inc, incision; SI, sleep deprivation combined with incision; TFUS, transcranial focused ultrasound; POD 7, postoperative day 7; TFUS-ExN, TFUS-responsive excitatory neurons; TRPC5, transient receptor potential (TRP) channel 5; IEGs, immediate-early genes. *P < 0.05; **P < 0.01; ***P < 0.001.

While the normalization of neural oscillations explains the network-level recovery, the sustained analgesic effect of 7 consecutive days of TFUS suggests underlying molecular and structural remodeling in the mPFC. Furthermore, the specific mechanosensor on mPFC^Glu^ neurons responsible for transducing the acoustic wave remains unknown. To explore this cellular-level mechanism, we performed single-nucleus RNA sequencing (snRNA-seq) on mPFC tissue from Inc+Sham, SI+Sham, and SI+TFUS mice (Fig. 5A), obtaining 77,477 nuclei post-quality control. Dimensionality reduction and graph-based clustering revealed 41 cellular clusters (Fig. 5B), which were subsequently annotated into 8 major cell types using canonical marker genes (Fig. 5C and D). The proportions of these major cell types showed no significant differences among the 3 groups (Fig. S10A and B).

Given the functional heterogeneity among mPFC^Glu^ subpopulations, we aimed to pinpoint those specifically mediating TFUS effects. Our analysis revealed 12 distinct mPFC^Glu^ subclusters (Fig. 5E). By assessing the expression of immediate-early genes (IEGs; Fos, Egr1, Arc, Junb, Nr4a1, and Dusp1), we identified 10 IEG-responsive subclusters where expression was suppressed in SI mice but robustly restored following TFUS. We defined these as TFUS-responsive excitatory neurons (TFUS-ExN) (Fig. 5E and F). Because ultrasound neuromodulation primarily acts through mechanosensitive ion channels (such as the KCNK, SCNN, PIEZO, and TRP families), we systematically screened ion channel expression. Focusing on the top 10 channels highly expressed in excitatory neurons with low inhibitory expression (Fig. 5G), we found that TRPC5 exhibited the highest expression specifically within the TFUS-ExN (Fig. 5H and I), highlighting it as a strong candidate for mediating mPFC ultrasound responses (Fig. 5J).

**Fig. 6.**
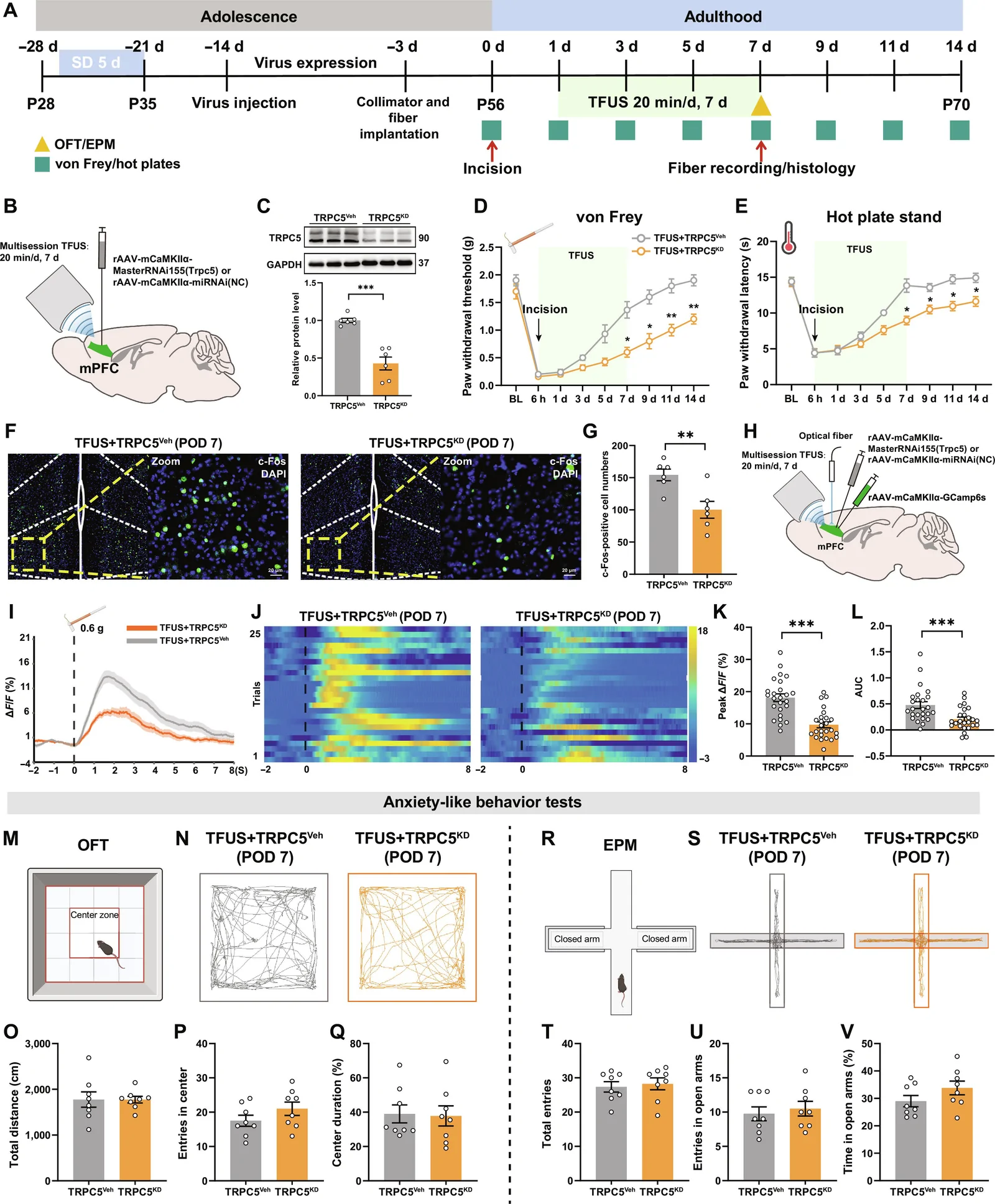
TRPC5 is an ultrasound-sensitive ion channel implicated in the therapeutic effects of TFUS. (A) Similar to Fig. 3A but preceded by mPFC injection of shRNA for TRPC5 knockdown prior to 200-Hz PRF TFUS stimulation in SI mice. On POD 7, behavioral testing and fiber photometry recordings were performed in separate cohorts of mice 3 h after the final TFUS or sham stimulation. Brain tissues for histological analysis were collected 1 h after the behavioral tests. (B) Schematic presentation of the viral injection and TFUS stimulation protocol. (C) Western blot evaluation of TRPC5 knockdown efficiency (*n* = 6; unpaired *t* test). (D and E) Time course assessment of nociceptive thresholds (*n* = 6; 2-way repeated-measures analysis of variance [ANOVA] with post hoc comparison). (F and G) c-Fos expression levels in mPFC (*n* = 6, unpaired *t* test). Scale bar, 20 μm. (H) Schematics of fiber photometry recording in TRPC5^KD^ or TRPC5^Veh^ mice following 7-d TFUS stimulation. (I and J) Heatmaps and the representative average Δ*F*/*F* traces (*n* = 5 mice per group, with 5 trials conducted per mouse). (K and L) Statistics of peak amplitudes (unpaired *t* test) and AUC (Mann–Whitney test) between changes across mice. (M to V) Behavioral tests, including the OFT and EPM, showing that TRPC5 knockdown in the mPFC followed by TFUS stimulation does not induce anxiety-like behaviors in SI mice (*n* = 8; unpaired *t* test). shRNA, short hairpin RNA; BL, baseline; AUC, area under the curve; mPFC, medial prefrontal cortex; SI, sleep deprivation combined with incision; TFUS, transcranial focused ultrasound; PRF, pulse repetition frequency; POD 7, postoperative day 7; TRPC5, transient receptor potential channel 5; TRPC5^KD^, TRPC5 knockdown; TRPC5^Veh^, TRPC5 vehicle control; OFT, open-field test; EPM, elevated plus maze. Data are presented as mean ± standard error of the mean (SEM). *P < 0.05; **P < 0.01; ***P < 0.001.

To directly confirm the functional activity of TRPC5 during ultrasound stimulation, we recorded the calcium signals of mPFC glutamatergic neurons using in vivo fiber photometry. Local microinjection of the selective TRPC5 inhibitor AC1903 significantly attenuated the acute calcium transients evoked by a single-session TFUS (200-Hz PRF) compared to vehicle controls (Fig. S11A to C). This demonstrates that TRPC5 activity mediates TFUS-induced neuronal excitation. Given the lack of cell-type specificity of pharmacological inhibition, we next used an adeno-associated virus (AAV) vector to specifically knock down TRPC5 expression in the mPFC^Glu^ of SI mice to further investigate its causal role in TFUS-mediated analgesia (Fig. 6A and B), with efficiency confirmed by Western blot (Fig. 6C). In TRPC5 knockdown (TRPC5^KD^) mice, the therapeutic efficacy of TFUS was markedly blunted. Compared to TRPC5 vehicle controls (TRPC5^Veh^), TRPC5^KD^ mice exhibited significantly lower mechanical (PWT) and thermal (PWL) pain thresholds on POD 7 (Fig. 6D and E). Notably, these threshold reductions on PODs 7, 9, and 14 were less pronounced than the overall pain improvements conferred by TFUS in SI mice (Fig. 3C and D). This indicates that TRPC5 knockdown only partially reverses TFUS effects, suggesting the involvement of additional ion channels or network dynamics.

Furthermore, this partial reversal was supported at the cellular level, as overall c-Fos expression (Fig. 6F and G) and the specific density of c-Fos^+^/CaMKIIα^+^ neurons (Fig. S11D and E) were significantly reduced in TRPC5^KD^ mice compared to that in TRPC5^Veh^ mice, with no changes observed in c-Fos^+^/GAD67^+^ neurons (Fig. S11D to F). Additionally, pain-evoked excitatory neuronal Ca^2+^ signal amplitudes (Fig. 6H to L) were significantly reduced in TRPC5^KD^ mice. Behavioral tests confirmed that these results were not confounded by anxiety or locomotor deficits (Fig. 6M to V). Collectively, these findings establish TRPC5 as a key molecular transducer that facilitates the specific ultrasonic activation of mPFC^Glu^ neurons to alleviate PPSP.

**Fig. 7.**
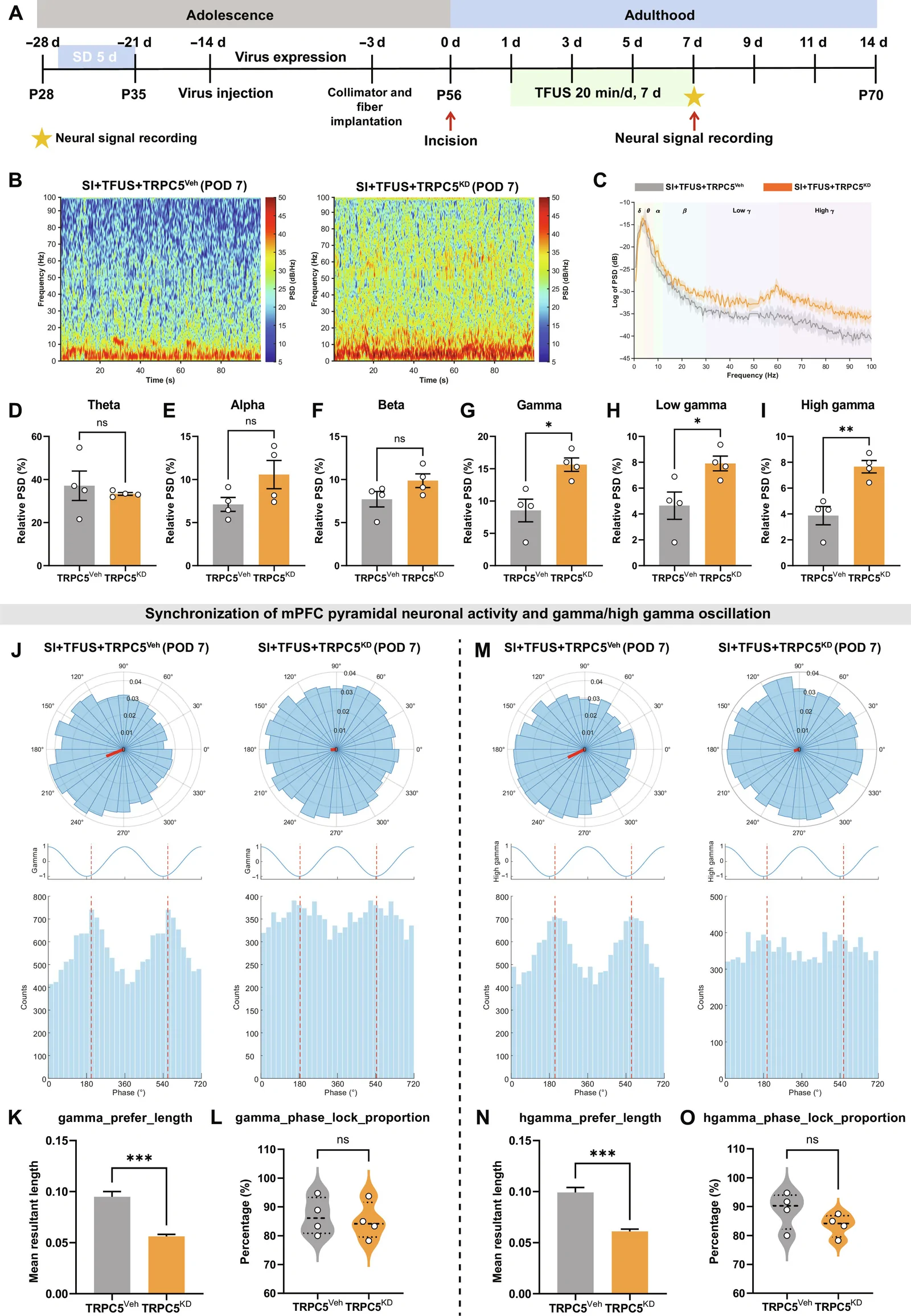
TRPC5 knockdown prevents TFUS-induced normalization of local network oscillations. (A) Timeline of experimental procedures. On POD 7, in vivo electrophysiological recordings were performed 3 h after the final 200-Hz PRF TFUS or sham stimulation. (B) Time–frequency representation of spectral power in the mPFC for the SI+TFUS+TRPC5^Veh^ and SI+TFUS+TRPC5^KD^ groups (*n* = 4). (C to I) Comparison of relative PSDs across frequency bands between the 2 groups (unpaired *t* test). (J) Representative plots of mPFC spike phase locking to local gamma oscillations. (K) The strength of mPFC neuron phase locking to local gamma oscillations (Mann–Whitney test). (L and O) The proportion of phase-locking mPFC neurons. (M) Representative plots of mPFC spike phase locking to local high-gamma oscillations. (N) The strength of mPFC neuron phase locking to local high-gamma oscillations (Mann–Whitney test). mPFC, medial prefrontal cortex; SI, sleep deprivation combined with incision; TFUS, transcranial focused ultrasound; PRF, pulse repetition frequency; POD 7, postoperative day 7; TRPC5, transient receptor potential channel 5; TRPC5^KD^, TRPC5 knockdown; TRPC5^Veh^, TRPC5 vehicle control; PSDs, power spectral densities. Data are presented as mean ± standard error of the mean (SEM). *P < 0.05; **P < 0.01; ***P < 0.001.

To determine whether the previously observed gamma normalization is a downstream consequence of TRPC5 activation, we performed in vivo electrophysiological recordings in TRPC5^KD^ mice (Fig. 7A). Compared to vehicle controls, TRPC5 knockdown prevented the TFUS-induced restoration of local network oscillations. Specifically, TRPC5^KD^ mice maintained significantly elevated relative gamma power (including both low and high bands) despite TFUS intervention (Fig. 7B to I). Furthermore, TRPC5 knockdown blocked the recovery of spike–gamma (Fig. 7J to L) and spike–high gamma phase locking (Fig. 7M to O). These results demonstrate that TRPC5 is required for TFUS-mediated gamma normalization, linking the initial mechanosensation to local network rhythm recovery.

### TFUS restores glutamatergic synaptic plasticity via the TRPC5–Homer1 pathway

**Fig. 8.**
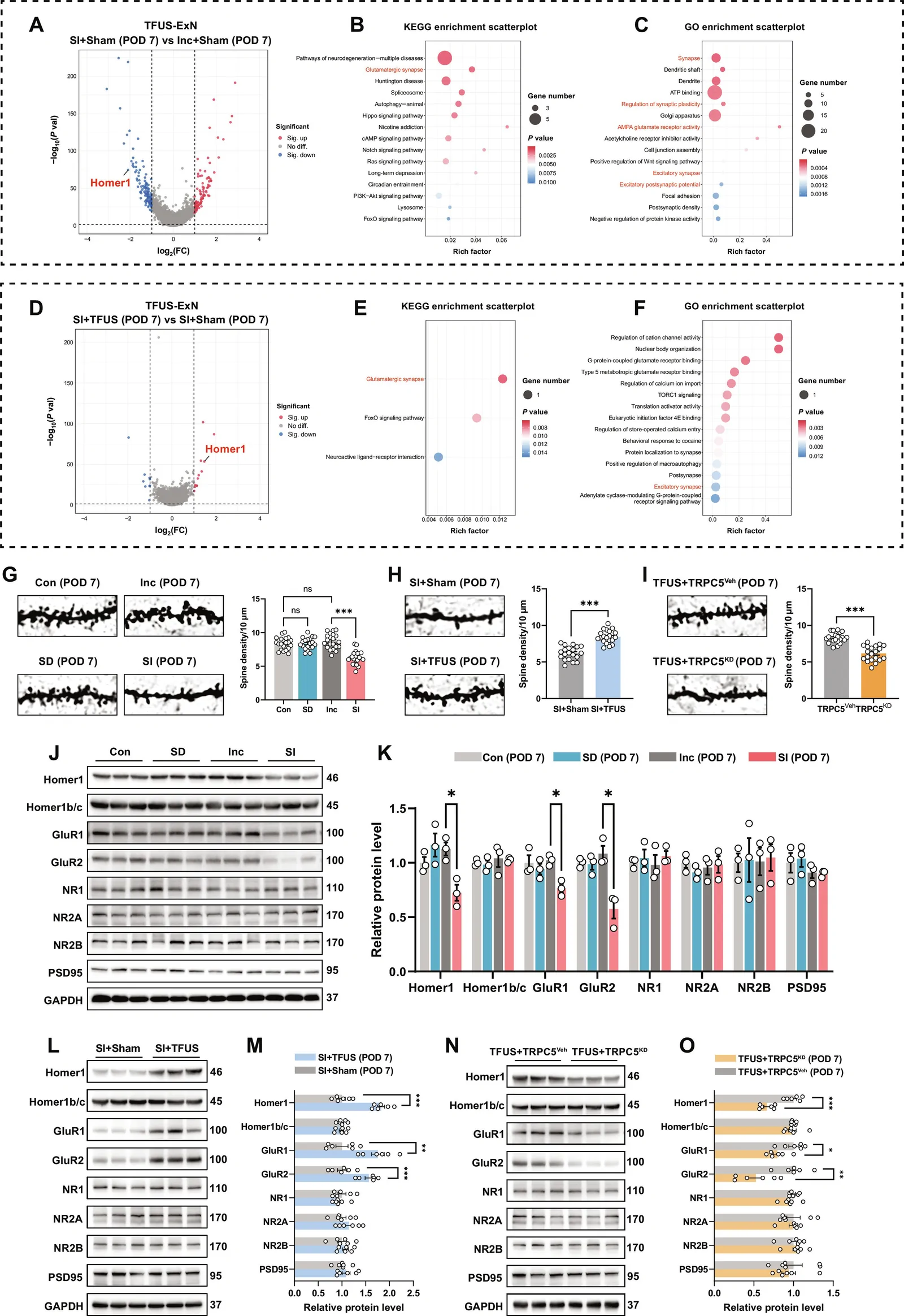
TFUS restores glutamatergic synaptic plasticity via the Homer1/AMPAR pathway in the mPFC. (A and D) Comparative analysis of Homer1 expression by volcano plot across experimental groups (SI+Sham vs Inc+Sham; SI+TFUS vs SI+Sham) in TFUS-ExN. Red represents relative increased expression DEGs, and blue indicates relative decreased expression DEGs. (B and C) Enrichment of KEGG pathways and GO terms from the SI+Sham vs Inc+Sham DEGs. (E and F) Enrichment of KEGG pathways and GO terms from the SI+TFUS vs SI+Sham DEGs. (G to I) Representative images and quantification of the dendritic spine density of mPFC pyramidal neurons (*n* = 20 neurons from 4 mice per group, unpaired *t* test). (J to O) Representative Western blots and quantitative data of Homer1, Homer1b/c, PSD95, and glutamate receptor (GluA1/2, NR2A/2B, and NR1) levels normalized to GAPDH in mPFC (*n* = 6, unpaired *t* test). AMPAR, α-amino-3-hydroxy-5-methyl-4-isoxazolepropionic acid receptor; TFUS-ExN, TFUS-responsive excitatory neurons; Con, control; SD, sleep deprivation; Inc, incision; SI, sleep deprivation combined with incision; TFUS, transcranial focused ultrasound; POD 7, postoperative day 7; TRPC5, transient receptor potential channel 5; TRPC5^KD^, TRPC5 knockdown; TRPC5^Veh^, TRPC5 vehicle control; DEGs, differentially expressed genes; KEGG, Kyoto Encyclopedia of Genes and Genomes; GO, Gene Ontology. Data are presented as mean ± standard error of the mean (SEM). *P < 0.05; **P < 0.01; ***P < 0.001.

Having established the critical role of TRPC5 as the primary mechanosensor in mediating both cellular- and network-level responses, we investigated the downstream gene regulatory mechanisms and structural changes underlying TFUS treatment. Differential gene expression analysis (differentially expressed genes) of TFUS-ExN and global excitatory neurons revealed distinct transcriptomic patterns across the Inc+Sham, SI+Sham, and SI+TFUS groups (Fig. 8A to F and Fig. S10C to H). Notably, for both injury-induced changes (SI+Sham vs Inc+Sham; Fig. 8B and C and Fig. S10D and E) and recovery-related changes (SI+TFUS vs SI+Sham; Fig. 8E and F and Fig. S10G and H), Kyoto Encyclopedia of Genes and Genomes and Gene Ontology analyses consistently highlighted significant enrichment in glutamatergic synapse signaling. Crucially, intersection analysis identified Homer1 as the sole overlapping gene within this shared pathway. In TFUS-ExN, Homer1 expression was suppressed in SI+Sham mice but effectively restored by TFUS (Fig. 8A and D), a pattern consistently mirrored in global excitatory neurons (Fig. S10C and F). These results identify Homer1-dependent glutamatergic synapse signaling as a shared mechanism underlying both adolescent SD-induced adult PPSP and TFUS-mediated therapeutic effects.

Guided by these transcriptomic findings, we utilized Golgi staining to evaluate structural synaptic plasticity in the mPFC. We found that dendritic spine density was comparable among the Con, SD, and Inc groups. However, compared to the Inc group, SI mice exhibited a significant decrease in dendritic spine density of approximately 28% (Fig. 8G), which was successfully reversed following TFUS treatment (Fig. 8H). To confirm whether this structural remodeling depends on TRPC5, we evaluated CaMKII-targeted TRPC5 knockdown mice and found that TRPC5^KD^ markedly blunted the TFUS-induced recovery of spine density (Fig. 8I). Finally, we characterized the corresponding molecular alterations by quantifying key synaptic proteins. Similarly, the baseline levels of these proteins were unaltered among the Con, SD, and Inc groups. In contrast, the SI group displayed significant reductions in Homer1 and α-amino-3-hydroxy-5-methyl-4-isoxazolepropionic acid receptor (AMPAR) subunits (GluA1 and GluA2) relative to the Inc group (Fig. 8J and K), which were effectively restored by 200-Hz PRF TFUS (Fig. 8L and M). Consistent with our morphological data, this TFUS-induced molecular up-regulation was largely abolished in TRPC5^KD^ mice (Fig. 8N and O). Meanwhile, the expression levels of Homer1b/c, NMDAR subunits (NR1 and NR2A/2B), and PSD95 remained unchanged across all groups (Fig. 8J to O), pinpointing AMPARs as the primary effectors. To further determine whether these proteins are functionally integrated into the synapses, we isolated synaptosomal fractions from the mPFC. Consistent with the total protein changes, the synaptic enrichment of Homer1, GluA1, and GluA2—quantified as ratios to PSD95—was significantly reduced in the SI group compared to that in the Inc group (Fig. S12A to E). Furthermore, TFUS treatment effectively restored the synaptic enrichment of these proteins, and this recovery was prevented by TRPC5 knockdown (Fig. S12F to J). Next, we directly tested the causal role of Homer1 by overexpressing it specifically in the mPFC^Glu^ of SI mice using an AAV vector (AAV-CaMKIIα-Homer1) in the absence of TFUS. We found that Homer1 overexpression alone rescued the dendritic spine density deficits (Fig. S13C and D). Furthermore, this intervention significantly alleviated PPSP, restoring both mechanical and thermal pain thresholds to baseline levels by POD 7 (Fig. S13A and B).

Collectively, these results demonstrate that Homer1–AMPAR down-regulation drives the impairment of spine density in PPSP mice. Conversely, TFUS acts through TRPC5 to restore glutamatergic synaptic plasticity and consolidate the multidimensional recovery of the mPFC, ultimately alleviating PPSP.

## Discussion

Adolescence is a critical window of brain maturation characterized by extensive synaptic remodeling [5,35]. Adolescent SD is a growing global health issue with lasting impacts on pain perception, emotional, and physical well-being [36,37]. Our study reveals that SD during this sensitive window exerts long-lasting effects on mPFC^Glu^, increasing vulnerability to PPSP in adulthood. The mPFC has emerged as an evolutionarily conserved hub for top-down modulation of sensory and affective processes [38,39], including pain perception [40,41]. Its late maturation in the brain [42,43] renders it particularly vulnerable to environmental insults during adolescence, such as sleep loss [44]. This developmental vulnerability likely predisposes the mPFC to functional abnormalities following surgery in adulthood, serving as a key trigger for PPSP.

Clinically, PPSP affects up to 50% of patients [10] and frequently co-occurs with other comorbidities, severely impairing postoperative recovery. Current pharmacological strategies, especially opioid-based therapies, remain inadequate for treating PPSP due to limited efficacy and substantial risks, including nausea, vomiting, addiction, and overdose [5,45]. This highlights an urgent need for innovative, nonpharmacological interventions [18,46]. In our study, fMRI comparisons between the Inc and SI groups identified the mPFC as the critical brain region mediating PPSP. Corroborating our fMRI results, previous studies have successfully targeted the mPFC for analgesia using noninvasive brain stimulation techniques, such as repetitive transcranial magnetic stimulation [47], transcranial direct current stimulation [48], and transcranial temporal interference stimulation [49]. However, these conventional techniques are often limited by low spatial resolution and poor targeting specificity. Therefore, in this context, we selected TFUS to modulate the mPFC, given its highly precise and region-specific modulation capabilities [50,51].

However, current TFUS applications exhibit substantial variability in parameters, leaving it unclear whether behavioral effects stem from global activation or selective network modulation [27,51,52]. In both clinical practice and animal research, the frequency of stimulation is considered a primary determinant of the direction of TFUS-induced neuromodulation [31,53]. Addressing this gap, we show that TFUS regulates pain-like behaviors in a frequency-dependent manner. Specifically, 200-Hz PRF TFUS selectively enhanced mPFC^Glu^ activity and ameliorated PPSP, whereas the 40- and 1,000-Hz PRF protocols were ineffective or showed limited efficacy. By successfully reversing mPFC^Glu^ hypoactivity, this finding underscores the necessity of establishing brain region- and symptom-specific TFUS protocols. While our study confirms the cellular-level efficacy of TFUS, the precise neurobiological and molecular mechanisms driving this targeted neuromodulation warrant further investigation at the network and molecular levels.

At the network level, previous research showed that gamma oscillations are critically implicated in pain perception [54,55]. Notably, gamma activation in S1 has been mechanistically linked to formalin-evoked nociception and noxious thermal stimuli [56], with its power scaling alongside inflammatory pain severity to encode persistent pain intensity [56,57]. In line with these observations, our data reveal a parallel phenomenon in the mPFC, where elevated gamma power strongly correlates with the manifestation of PPSP. Furthermore, in the SI group, mPFC pyramidal neurons exhibited a marked reduction in phase locking to local gamma rhythms, indicating impaired nociceptive processing and integration. This disruption in neural oscillations likely results from a local excitation–inhibition imbalance driven by mPFC^Glu^ hypoactivity. Building on this, we propose that this disruption in spike–gamma phase coupling underlies the inability of the mPFC to effectively integrate nociceptive signals for analgesia. Interestingly, TFUS intervention not only attenuated mPFC gamma power but also restored spike–gamma phase coupling. These findings provide the first evidence that TFUS ameliorates PPSP through the targeted modulation of network-level oscillatory dynamics, thereby establishing a neuro-oscillatory framework for its therapeutic mechanisms.

Beyond network dynamics, we further employed snRNA-seq to elucidate the molecular and structural basis of TFUS neuromodulation. Our analysis identified TRPC5 as a key ultrasound-sensitive ion channel highly enriched in TFUS-ExN, suggesting that TFUS may achieve its therapeutic effects by coordinating the activity of pain-regulatory networks within the TFUS-ExN populations. Crucially, our findings link this TRPC5 activation directly to the restoration of structural synaptic plasticity. The observed mPFC^Glu^ hypoactivity is not an isolated functional defect but is fundamentally rooted in the loss of dendritic spines mediated by the Homer1–AMPAR pathway. Besides TRPC5, other TRP and PIEZO channel family members were also broadly expressed in mPFC^Glu^ neurons. Consistent with the broad expression of other mechanosensitive channels (e.g., PIEZO), TRPC5 knockdown only partially attenuated the ultrasound-induced effects, indicating the involvement of parallel mechanisms in TFUS-mediated pain relief [58]. Supporting this hypothesis, our behavioral results revealed that application of GsMTx4, a broad-spectrum inhibitor that blocks both PIEZO and TRPC channels, effectively abolished the TFUS-induced analgesia (Fig. S14A and B). This confirms that while TRPC5 is a key mediator, other mechanosensitive channels such as PIEZO also contribute to the overall therapeutic effect. Future studies should explore the interplay between these channels to fully delineate the molecular landscape of TFUS.

Translating these findings into clinical practice requires addressing patient identification, therapeutic timing, and safety. Given that routine analgesia is often insufficient for patients with sleep deficiency-induced pain susceptibility, preoperative assessments could incorporate sleep questionnaires (e.g., Pittsburgh Sleep Quality Index) to identify at-risk individuals. For these patients, TFUS could be applied during the early postoperative phase to disrupt the transition from acute to chronic pain. Furthermore, to account for the thicker human skull, clinical TFUS typically utilizes lower frequencies and MRI neuronavigation to safely target deep regions. By maintaining acoustic parameters below FDA safety limits, this approach offers a feasible noninvasive strategy for preventing PPSP.

This study has several potential limitations. First, although both sexes exhibited SD-induced PPSP vulnerability, our mechanistic experiments were restricted to male mice. Adolescent female mice have smaller skulls and lower body weights, making it technically challenging to support the heavy head-mounted apparatus required for freely moving electrophysiology and ultrasound stimulation without inducing physical stress. Thus, whether the observed neural mechanisms fully apply to females remains to be investigated. Second, we propose that TFUS-induced analgesia may be driven by mPFC-mediated top-down inhibition. While our chemogenetic experiments establish a causal link between local mPFC^Glu^ activity and pain behavior, the exact downstream circuit mechanisms remain to be fully elucidated. Future studies employing pathway-specific interventions would help validate the complete circuit, such as the projection from the mPFC to the PAG. Third, the plantar incision model produces relatively mild tissue injury compared to major clinical procedures. Although it effectively models how preoperative vulnerabilities predispose individuals to PPSP following minor surgeries, it may not fully capture mechanisms driven by severe nerve damage. Future studies utilizing more severe injury models would help evaluate the generalizability of our findings. Fourth, the spontaneous recovery of pain thresholds in our incision model limits the precise evaluation of long-term analgesic persistence and late-stage TFUS efficacy. Future studies using nonrecovering chronic pain models would help clarify these temporal dynamics and further optimize prophylactic protocols. Finally, while our automated protocol minimizes physical exertion, the prolonged intervention inherently introduces mild stress and activity. Thus, the observed PPSP phenotype likely reflects the combined effects of sleep loss and these concurrent factors.

## Conclusion

In conclusion, our study establishes an adult PPSP model induced by adolescent SD and identifies mPFC^Glu^ hypoactivity as a central mediator of this condition. This reduced mPFC^Glu^ excitability likely desynchronizes these neurons from the local network, generating aberrant neural oscillations. TFUS alleviates this condition through an integrated mechanistic pathway: activating TRPC5 channels to promote Homer1-dependent synaptic plasticity, which subsequently reverses mPFC^Glu^ hypoactivity and normalizes local neural rhythms (Fig. S15). By linking molecular mechanosensation with the restoration of cellular excitability and network synchronization, our findings highlight the therapeutic potential of TFUS as a noninvasive intervention for PPSP.

## Materials and Methods

### Animals

C57BL/6J mice (3 to 4 weeks, male and female) were supplied by Beijing Vital River Laboratory and housed under controlled conditions (22 ± 1 °C, 40% to 60% humidity) on a 12-h light/dark cycle (lights on 0800 to 2000; ZT0 to ZT12), with free access to food and water. Female mice were used exclusively for the initial pain behavioral validation of the model. Male mice were utilized for both behavioral assessments and all subsequent mechanistic experiments (including in vivo electrophysiology, fiber photometry, TFUS interventions, and histological analyses). All experimental animals were randomly allocated into each experimental group.

### Ultrasound stimulation

The radio-frequency signals were generated by an ultrasound neurostimulation system (GreenValley, China) to drive an ultrasound transducer at a fundamental frequency (*f*_0_) of 2 MHz. To maintain a constant ultrasound DC of 10% across different PRFs, the tone-burst duration (TBD) and the number of cycles per pulse were adjusted accordingly. Specifically, for a PRF of 40 Hz, the TBD was 2.5 ms (5,000 cycles per pulse); for 200 Hz, the TBD was 0.5 ms (1,000 cycles per pulse); and for 1,000 Hz, the TBD was 0.1 ms (200 cycles per pulse). These ultrasound pulses were delivered within an ultrasound duration of 1 s, followed by an ISoI of 8 s. The acoustic fields, both original and transcranial, were characterized in a degassed water tank using a calibrated needle hydrophone (SN2791, 0.5-mm probe, Precision Acoustics, UK) and a 3D ultrasound intensity measurement system (UMS3, Precision Acoustics, UK). The propagation through the mouse skull and dura resulted in an acoustic pressure attenuation of approximately 36%. The transcranial focal spot measured 0.81 mm in width and 0.85 mm in length at the −3-dB level without the skull. In the presence of the mouse skull, the focal dimensions were 0.9 mm in width and 0.84 mm in length at −3 dB. The employed ultrasound intensity levels and DCs are described in Table S1. As noted in Table S1, the ultrasound parameters for those conditions were to keep the ultrasound DC constant at 10%. This set of parameters also maintained equivalent spatial-peak temporal-average intensity (*I*_SPTA_) and spatial-peak pulse-average intensity (*I*_SPPA_), respectively, among PRF levels.

### Other methods

Detailed protocols for SD, plantar incision, behavioral tests (von Frey, hot plate, OFT, and EPM), sleep electroencephalogram recording, fiber photometry, in vivo electrophysiology, fMRI acquisition, snRNA-seq, Golgi staining, Western blotting, and immunohistochemistry are available in the Supplementary Materials.

### Statistics and reproducibility

MATLAB or GraphPad Prism 10 (GraphPad Software, USA) was used for analyses and graphing. For all representative images and statistical analyses, experiments were independently replicated in at least 3 mice, yielding consistent results. This reproducibility was confirmed across both behavioral tests and fiber-photometric calcium recordings.

Data normality was assessed using the Shapiro–Wilk test. For comparisons between 2 groups, either parametric tests (paired or unpaired 2-tailed Student *t* tests) or their nonparametric equivalents (Mann–Whitney *U* test or Wilcoxon test) were applied based on the normality test outcome. For comparisons among 3 groups, a one-way analysis of variance (ANOVA) was used if the data met assumptions of both normality and homogeneity of variance; otherwise, the Kruskal–Wallis test was employed. For experimental designs involving multiple comparisons, a 2-way ANOVA followed by post hoc tests was utilized. Data are presented as mean ± standard error of the mean. Significance levels are denoted as *P < 0.05, **P < 0.01, and ***P < 0.001; *P* values below 0.0001 are not reported as exact values.

## Ethical Approval

All animal procedures were approved by the Animal Ethics Committee of the Affiliated Nanjing Drum Tower Hospital, Medical School of Nanjing University (Approval No. 2023AE01069), and followed the ARRIVE guidelines.

## Data Availability

The data that support the findings of this study are available from the corresponding authors upon reasonable request.
