# Supplementary material for: Targeting the Medial Prefrontal Cortex with Transcranial Focused Ultrasound Alleviates Adolescent Sleep-Deprivation-Induced Persistent Postsurgical Pain in Adulthood
Source: Research (Wash D C). 2026 Sep 23;9:1437. doi: 10.34133/research.1437 (PMC13598325; doi:10.34133/research.1437)
Supplement: Supplementary file 1 [file research.1437.f1.zip › FigureS1.pdf]

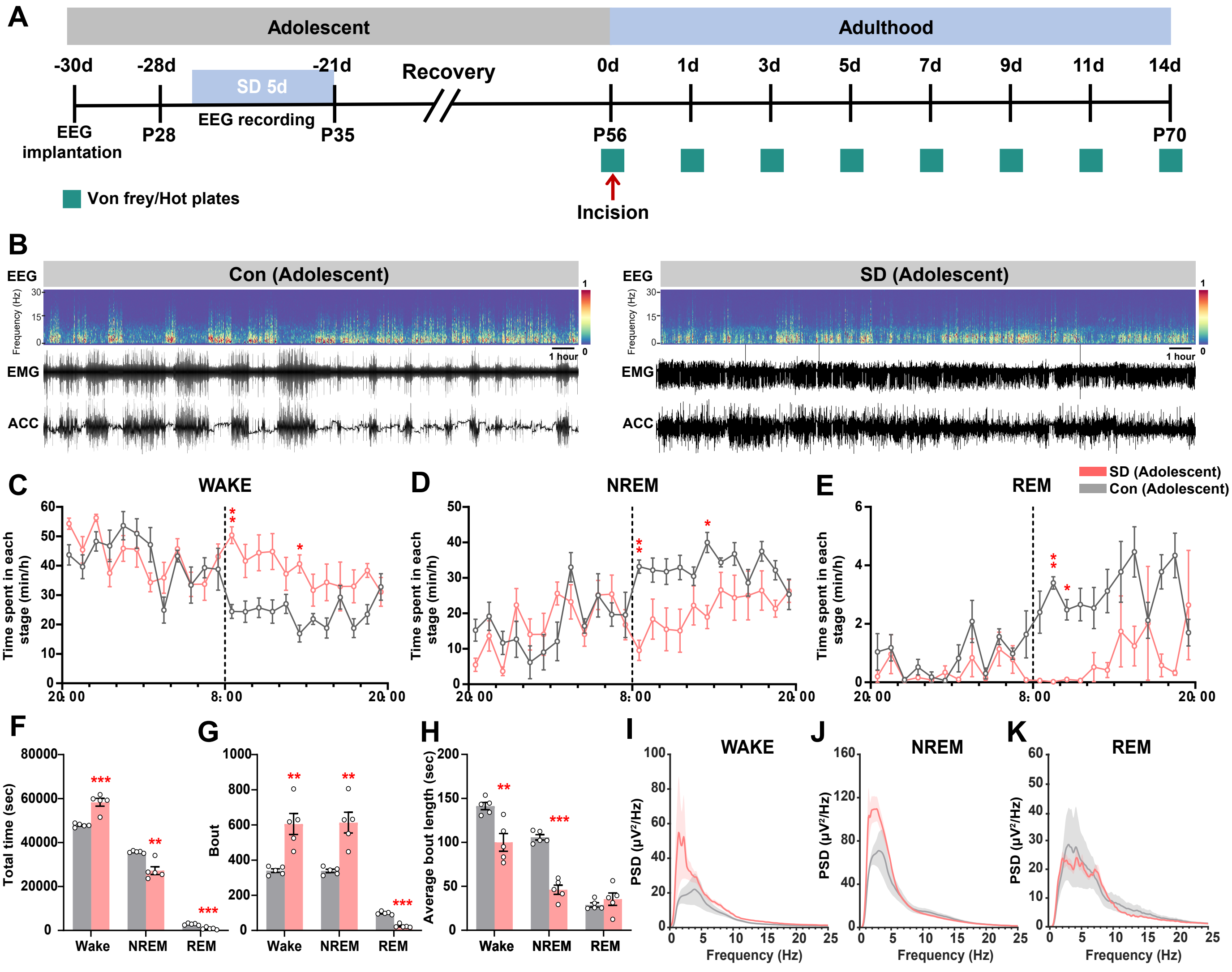
A
Adolescent
Adulthood
-30d
-28d
SD 5d
-21d
Recovery
0d
1d
3d
5d
7d
9d
11d
14d
EEG implantation
P28
EEG recording
P35
P56
P70
Von frey/Hot plates
Incision
B
EEG
Con (Adolescent)
SD (Adolescent)
Frequency (Hz)
1 hour
EMG
ACC
C
WAKE
D
NREM
E
REM
SD (Adolescent)
Con (Adolescent)
Time spent in each stage (min/h)
20: 00
8: 00
F
Total time (sec)
Wake
NREM
REM
G
Bout
H
Average bout length (sec)
I
WAKE
J
NREM
K
REM
PSD (μV²/Hz)
Frequency (Hz)
