# Supplementary material for: Targeting the Medial Prefrontal Cortex with Transcranial Focused Ultrasound Alleviates Adolescent Sleep-Deprivation-Induced Persistent Postsurgical Pain in Adulthood
Source: Research (Wash D C). 2026 Sep 23;9:1437. doi: 10.34133/research.1437 (PMC13598325; doi:10.34133/research.1437)
Supplement: Supplementary file 1 [file research.1437.f1.zip › FigureS10.pdf]

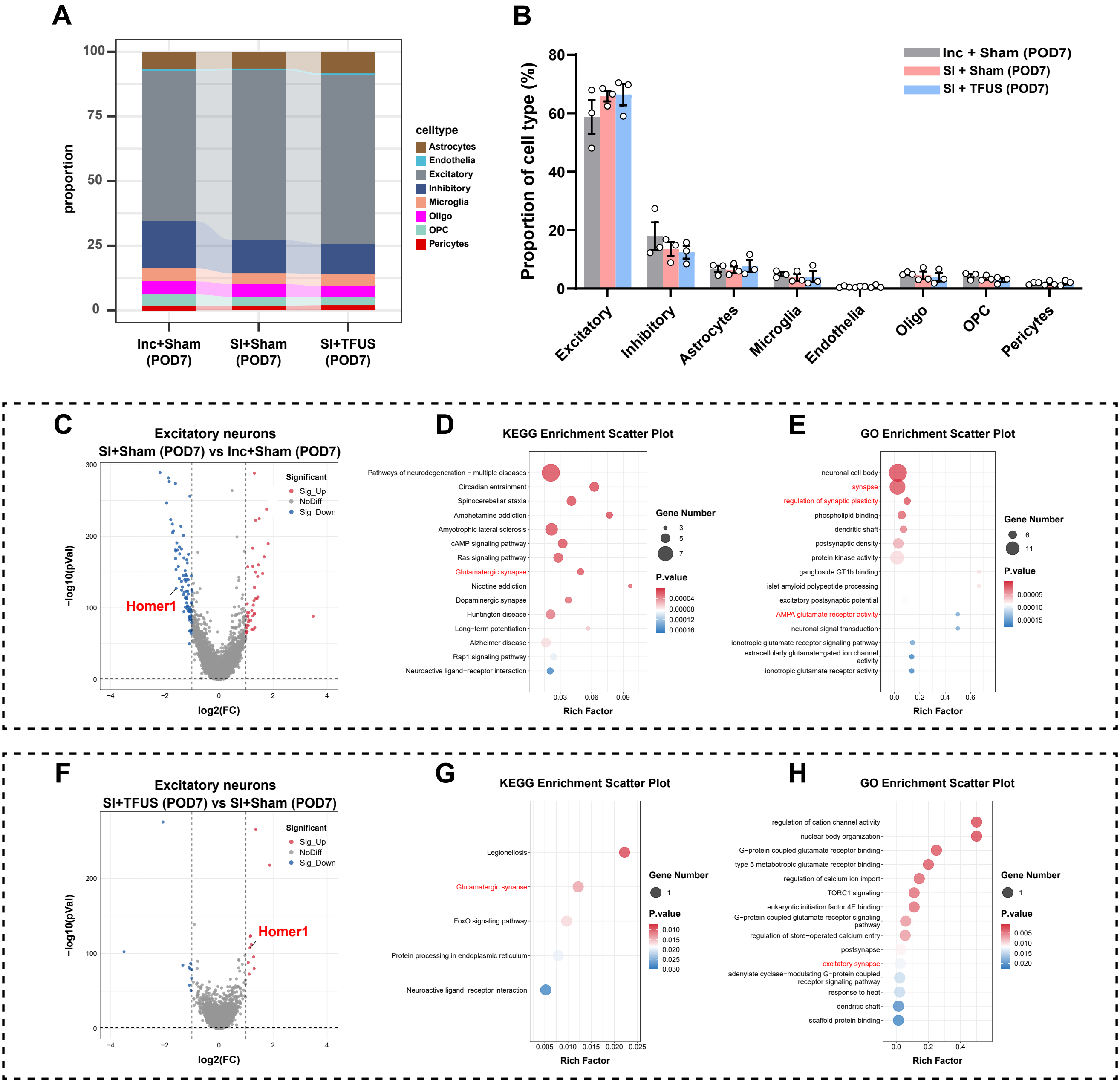
A
proportion
100
75
50
25
0
Inc+Sham (POD7)
SI+Sham (POD7)
SI+TFUS (POD7)
celltype
Astrocytes
Endothelia
Excitatory
Inhibitory
Microglia
Oligo
OPC
Pericytes
B
Proportion of cell type (%)
80
60
40
20
0
Inc + Sham (POD7)
SI + Sham (POD7)
SI + TFUS (POD7)
Excitatory
Inhibitory
Astrocytes
Microglia
Endothelia
Oligo
OPC
Pericytes
C
Excitatory neurons
SI+Sham (POD7) vs Inc+Sham (POD7)
−log10(pVal)
log2(FC)
Significant
Sig_Up
NoDiff
Sig_Down
Homer1
D
KEGG Enrichment Scatter Plot
Pathways of neurodegeneration − multiple diseases
Circadian entrainment
Spinocerebellar ataxia
Amphetamine addiction
Amyotrophic lateral sclerosis
cAMP signaling pathway
Ras signaling pathway
Glutamatergic synapse
Nicotine addiction
Dopaminergic synapse
Huntington disease
Long−term potentiation
Alzheimer disease
Rap1 signaling pathway
Neuroactive ligand−receptor interaction
Rich Factor
Gene Number
P.value
E
GO Enrichment Scatter Plot
neuronal cell body
synapse
regulation of synaptic plasticity
phospholipid binding
dendritic shaft
postsynaptic density
protein kinase activity
ganglioside GT1b binding
islet amyloid polypeptide processing
excitatory postsynaptic potential
AMPA glutamate receptor activity
neuronal signal transduction
ionotropic glutamate receptor signaling pathway
extracellularly glutamate−gated ion channel activity
ionotropic glutamate receptor activity
Rich Factor
Gene Number
P.value
F
Excitatory neurons
SI+TFUS (POD7) vs SI+Sham (POD7)
−log10(pVal)
log2(FC)
Significant
Sig_Up
NoDiff
Sig_Down
Homer1
G
KEGG Enrichment Scatter Plot
Legionellosis
Glutamatergic synapse
FoxO signaling pathway
Protein processing in endoplasmic reticulum
Neuroactive ligand−receptor interaction
Rich Factor
Gene Number
P.value
H
GO Enrichment Scatter Plot
regulation of cation channel activity
nuclear body organization
G−protein coupled glutamate receptor binding
type 5 metabotropic glutamate receptor binding
regulation of calcium ion import
TORC1 signaling
eukaryotic initiation factor 4E binding
G−protein coupled glutamate receptor signaling pathway
regulation of store−operated calcium entry
postsynapse
excitatory synapse
adenylate cyclase−modulating G−protein coupled receptor signaling pathway
response to heat
dendritic shaft
scaffold protein binding
Rich Factor
Gene Number
P.value
