# Supplementary material for: Targeting the Medial Prefrontal Cortex with Transcranial Focused Ultrasound Alleviates Adolescent Sleep-Deprivation-Induced Persistent Postsurgical Pain in Adulthood
Source: Research (Wash D C). 2026 Sep 23;9:1437. doi: 10.34133/research.1437 (PMC13598325; doi:10.34133/research.1437)
Supplement: Supplementary file 1 [file research.1437.f1.zip › FigureS11.pdf]

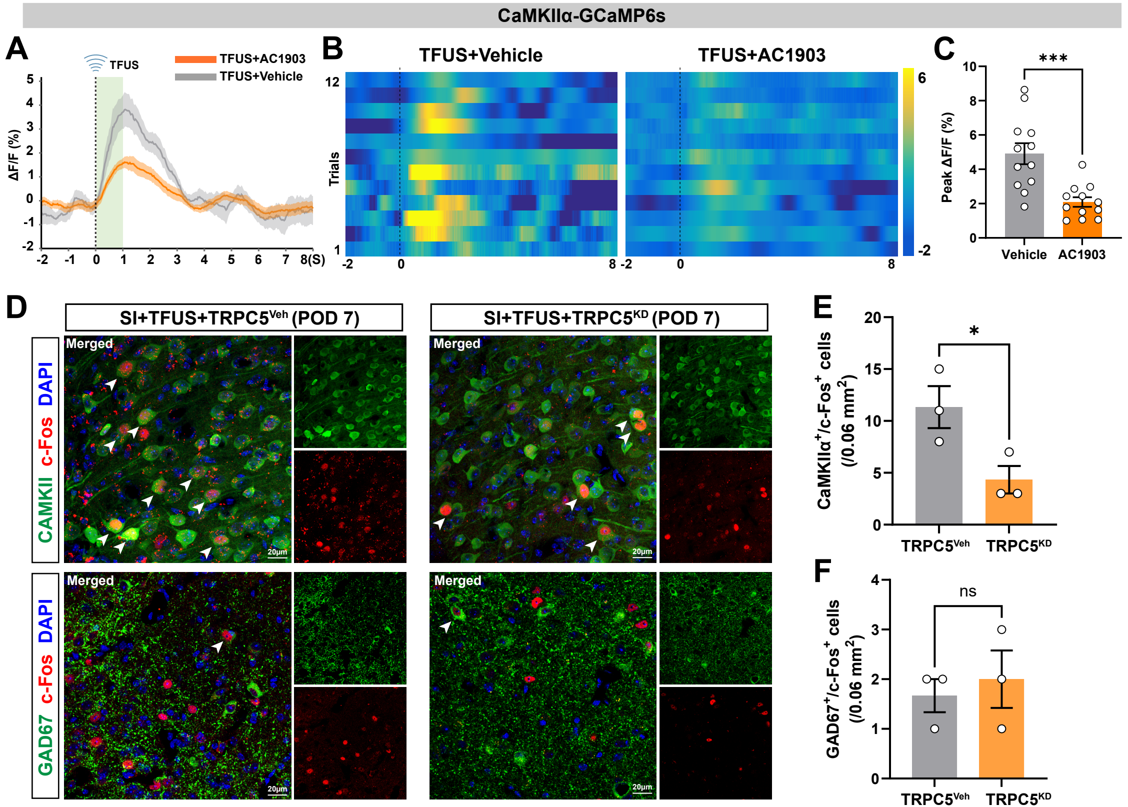

CaMKIIα-GCaMP6s
A
TFUS
TFUS+AC1903
TFUS+Vehicle
ΔF/F (%)
8(S)
B
TFUS+Vehicle
TFUS+AC1903
Trials
C
***
Peak ΔF/F (%)
Vehicle
AC1903
D
SI+TFUS+TRPC5Veh (POD 7)
SI+TFUS+TRPC5KD (POD 7)
Merged
CAMKII c-Fos DAPI
GAD67 c-Fos DAPI
20μm
E
*
CaMKIIα+/c-Fos+ cells (/0.06 mm2)
TRPC5Veh
TRPC5KD
F
ns
GAD67+/c-Fos+ cells (/0.06 mm2)
TRPC5Veh
TRPC5KD
