# Supplementary material for: Targeting the Medial Prefrontal Cortex with Transcranial Focused Ultrasound Alleviates Adolescent Sleep-Deprivation-Induced Persistent Postsurgical Pain in Adulthood
Source: Research (Wash D C). 2026 Sep 23;9:1437. doi: 10.34133/research.1437 (PMC13598325; doi:10.34133/research.1437)
Supplement: Supplementary file 1 [file research.1437.f1.zip › FigureS12.pdf]

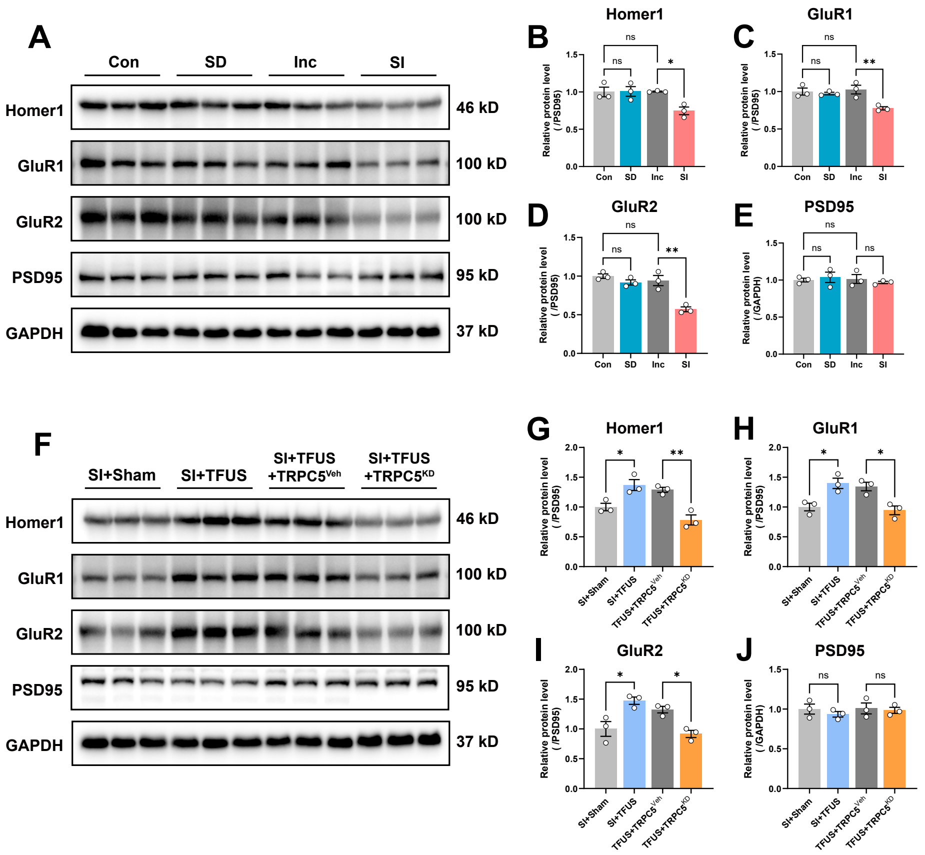

A
Con
SD
Inc
SI
Homer1
GluR1
GluR2
PSD95
GAPDH
46 kD
100 kD
100 kD
95 kD
37 kD
B
Homer1
C
GluR1
D
GluR2
E
PSD95
Relative protein level (/PSD95)
Relative protein level (/GAPDH)
ns
*
**
F
SI+Sham
SI+TFUS
SI+TFUS +TRPC5Veh
SI+TFUS +TRPC5KD
G
Homer1
H
GluR1
I
GluR2
J
PSD95
TFUS+TRPC5Veh
TFUS+TRPC5KD
