# Supplementary material for: Targeting the Medial Prefrontal Cortex with Transcranial Focused Ultrasound Alleviates Adolescent Sleep-Deprivation-Induced Persistent Postsurgical Pain in Adulthood
Source: Research (Wash D C). 2026 Sep 23;9:1437. doi: 10.34133/research.1437 (PMC13598325; doi:10.34133/research.1437)
Supplement: Supplementary file 1 [file research.1437.f1.zip › FigureS13.pdf]

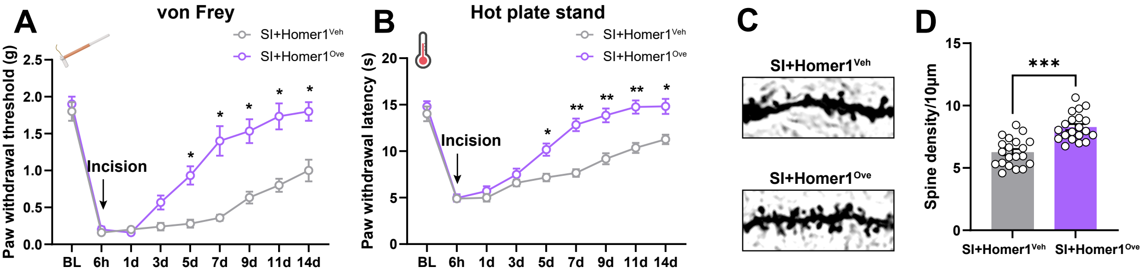

A
von Frey
SI+Homer1Veh
SI+Homer1Ove
Paw withdrawal threshold (g)
2.5
2.0
1.5
1.0
0.5
0.0
Incision
BL 6h 1d 3d 5d 7d 9d 11d 14d
B
Hot plate stand
SI+Homer1Veh
SI+Homer1Ove
Paw withdrawal latency (s)
20
15
10
5
0
Incision
BL 6h 1d 3d 5d 7d 9d 11d 14d
C
SI+Homer1Veh
SI+Homer1Ove
D
Spine density/10μm
15
10
5
0
***
SI+Homer1Veh
SI+Homer1Ove
