# Supplementary material for: Targeting the Medial Prefrontal Cortex with Transcranial Focused Ultrasound Alleviates Adolescent Sleep-Deprivation-Induced Persistent Postsurgical Pain in Adulthood
Source: Research (Wash D C). 2026 Sep 23;9:1437. doi: 10.34133/research.1437 (PMC13598325; doi:10.34133/research.1437)
Supplement: Supplementary file 1 [file research.1437.f1.zip › FigureS14.pdf]

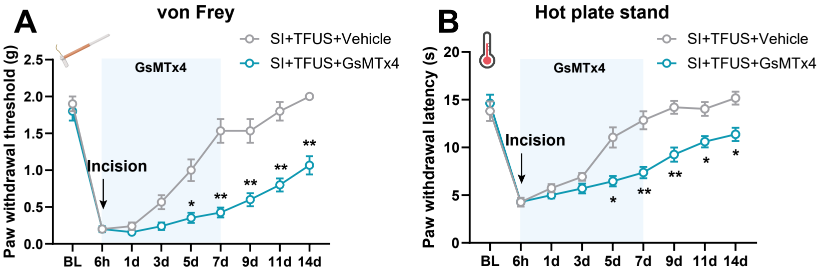

A
von Frey
SI+TFUS+Vehicle
SI+TFUS+GsMTx4
GsMTx4
Paw withdrawal threshold (g)
2.5
2.0
1.5
1.0
0.5
0.0
Incision
*
**
**
**
**
BL
6h
1d
3d
5d
7d
9d
11d
14d
B
Hot plate stand
SI+TFUS+Vehicle
SI+TFUS+GsMTx4
GsMTx4
Paw withdrawal latency (s)
20
15
10
5
0
Incision
*
**
**
*
*
BL
6h
1d
3d
5d
7d
9d
11d
14d
