# Supplementary material for: Targeting the Medial Prefrontal Cortex with Transcranial Focused Ultrasound Alleviates Adolescent Sleep-Deprivation-Induced Persistent Postsurgical Pain in Adulthood
Source: Research (Wash D C). 2026 Sep 23;9:1437. doi: 10.34133/research.1437 (PMC13598325; doi:10.34133/research.1437)
Supplement: Supplementary file 1 [file research.1437.f1.zip › FigureS15.pdf]

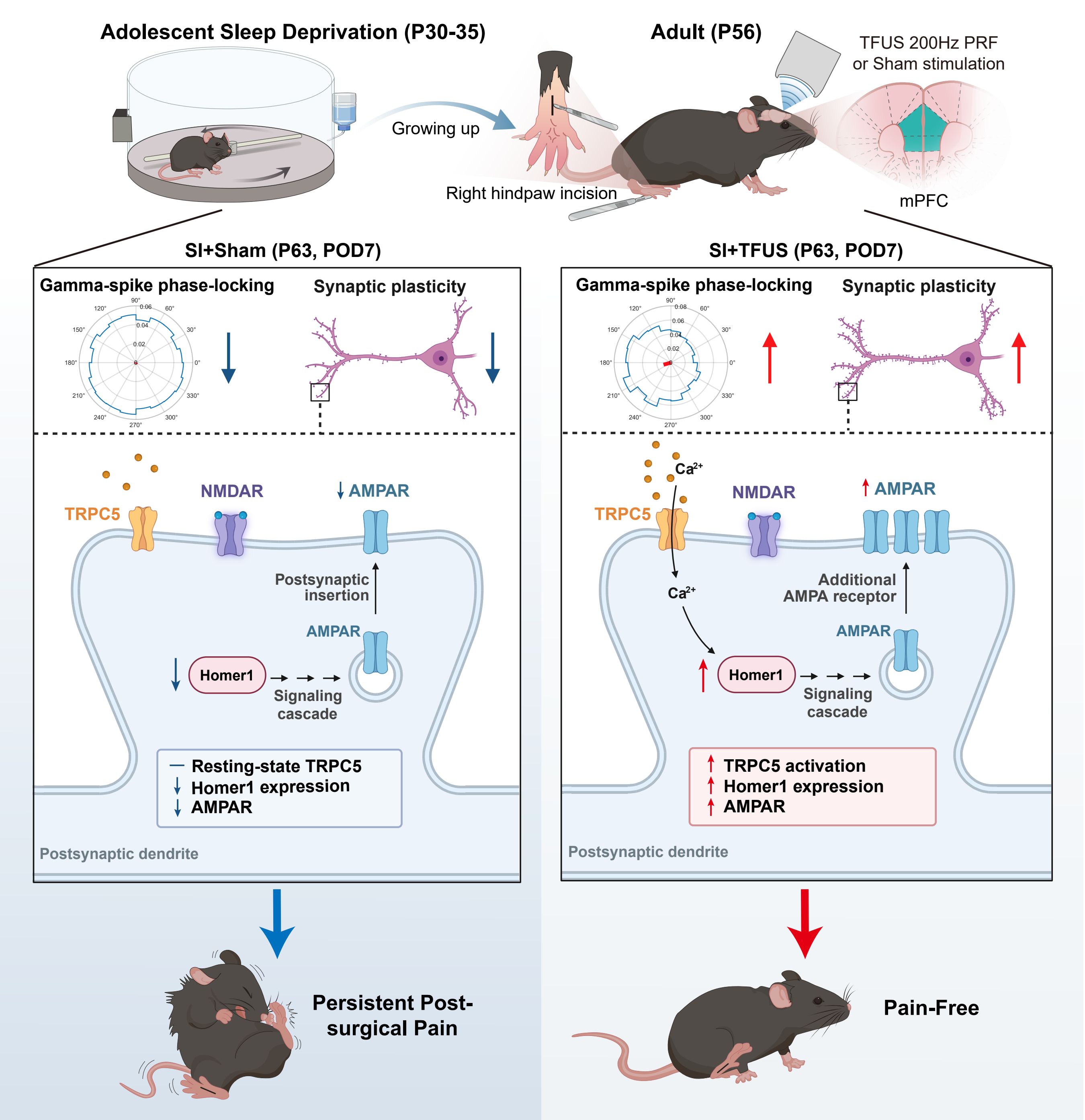

Adolescent Sleep Deprivation (P30-35)
Growing up
Adult (P56)
TFUS 200Hz PRF
or Sham stimulation
Right hindpaw incision
mPFC
SI+Sham (P63, POD7)
Gamma-spike phase-locking
Synaptic plasticity
TRPC5
NMDAR
AMPAR
Postsynaptic
insertion
AMPAR
Homer1
Signaling
cascade
— Resting-state TRPC5
↓ Homer1 expression
↓ AMPAR
Postsynaptic dendrite
SI+TFUS (P63, POD7)
Gamma-spike phase-locking
Synaptic plasticity
Ca2+
TRPC5
NMDAR
AMPAR
Ca2+
Additional
AMPA receptor
AMPAR
Homer1
Signaling
cascade
↑ TRPC5 activation
↑ Homer1 expression
↑ AMPAR
Postsynaptic dendrite
Persistent Post-surgical Pain
Pain-Free
