# Supplementary material for: Targeting the Medial Prefrontal Cortex with Transcranial Focused Ultrasound Alleviates Adolescent Sleep-Deprivation-Induced Persistent Postsurgical Pain in Adulthood
Source: Research (Wash D C). 2026 Sep 23;9:1437. doi: 10.34133/research.1437 (PMC13598325; doi:10.34133/research.1437)
Supplement: Supplementary file 1 [file research.1437.f1.zip › FigureS2.pdf]

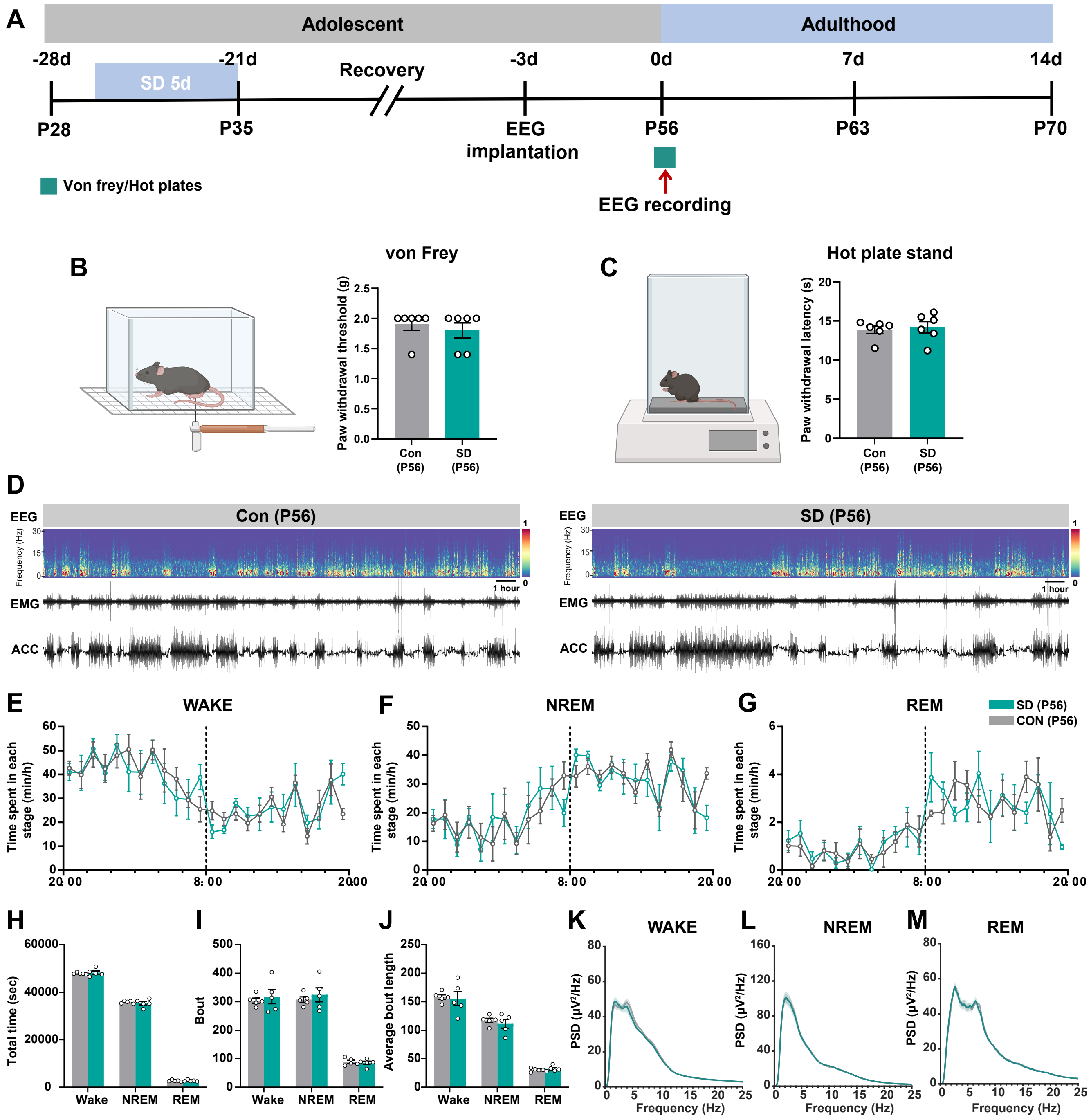

A
Adolescent
Adulthood
-28d
-21d
-3d
0d
7d
14d
SD 5d
Recovery
P28
P35
EEG implantation
P56
P63
P70
Von frey/Hot plates
EEG recording
B
von Frey
Paw withdrawal threshold (g)
Con (P56)
SD (P56)
C
Hot plate stand
Paw withdrawal latency (s)
Con (P56)
SD (P56)
D
EEG
Con (P56)
SD (P56)
Frequency (Hz)
1 hour
EMG
ACC
E
WAKE
F
NREM
G
REM
SD (P56)
CON (P56)
Time spent in each stage (min/h)
20: 00
8: 00
20: 00
H
Total time (sec)
I
Bout
J
Average bout length
Wake
NREM
REM
K
WAKE
L
NREM
M
REM
PSD (μV²/Hz)
Frequency (Hz)
