# Supplementary material for: Targeting the Medial Prefrontal Cortex with Transcranial Focused Ultrasound Alleviates Adolescent Sleep-Deprivation-Induced Persistent Postsurgical Pain in Adulthood
Source: Research (Wash D C). 2026 Sep 23;9:1437. doi: 10.34133/research.1437 (PMC13598325; doi:10.34133/research.1437)
Supplement: Supplementary file 1 [file research.1437.f1.zip › FigureS3.pdf]

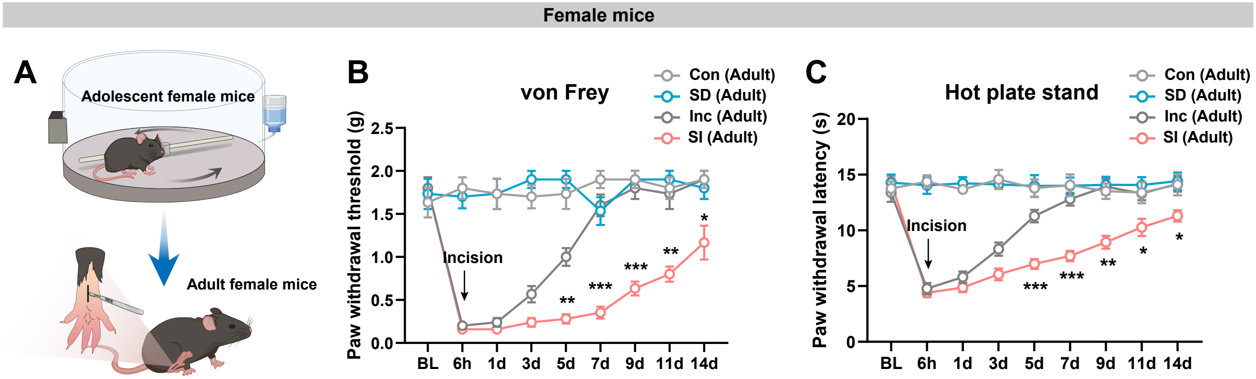

Female mice
A
Adolescent female mice
Adult female mice
B
von Frey
Con (Adult)
SD (Adult)
Inc (Adult)
SI (Adult)
Paw withdrawal threshold (g)
2.5
2.0
1.5
1.0
0.5
0.0
Incision
**
***
***
**
*
BL
6h
1d
3d
5d
7d
9d
11d
14d
C
Hot plate stand
Con (Adult)
SD (Adult)
Inc (Adult)
SI (Adult)
Paw withdrawal latency (s)
20
15
10
5
0
Incision
***
***
**
*
*
BL
6h
1d
3d
5d
7d
9d
11d
14d
