# Supplementary material for: Targeting the Medial Prefrontal Cortex with Transcranial Focused Ultrasound Alleviates Adolescent Sleep-Deprivation-Induced Persistent Postsurgical Pain in Adulthood
Source: Research (Wash D C). 2026 Sep 23;9:1437. doi: 10.34133/research.1437 (PMC13598325; doi:10.34133/research.1437)
Supplement: Supplementary file 1 [file research.1437.f1.zip › FigureS4.pdf]

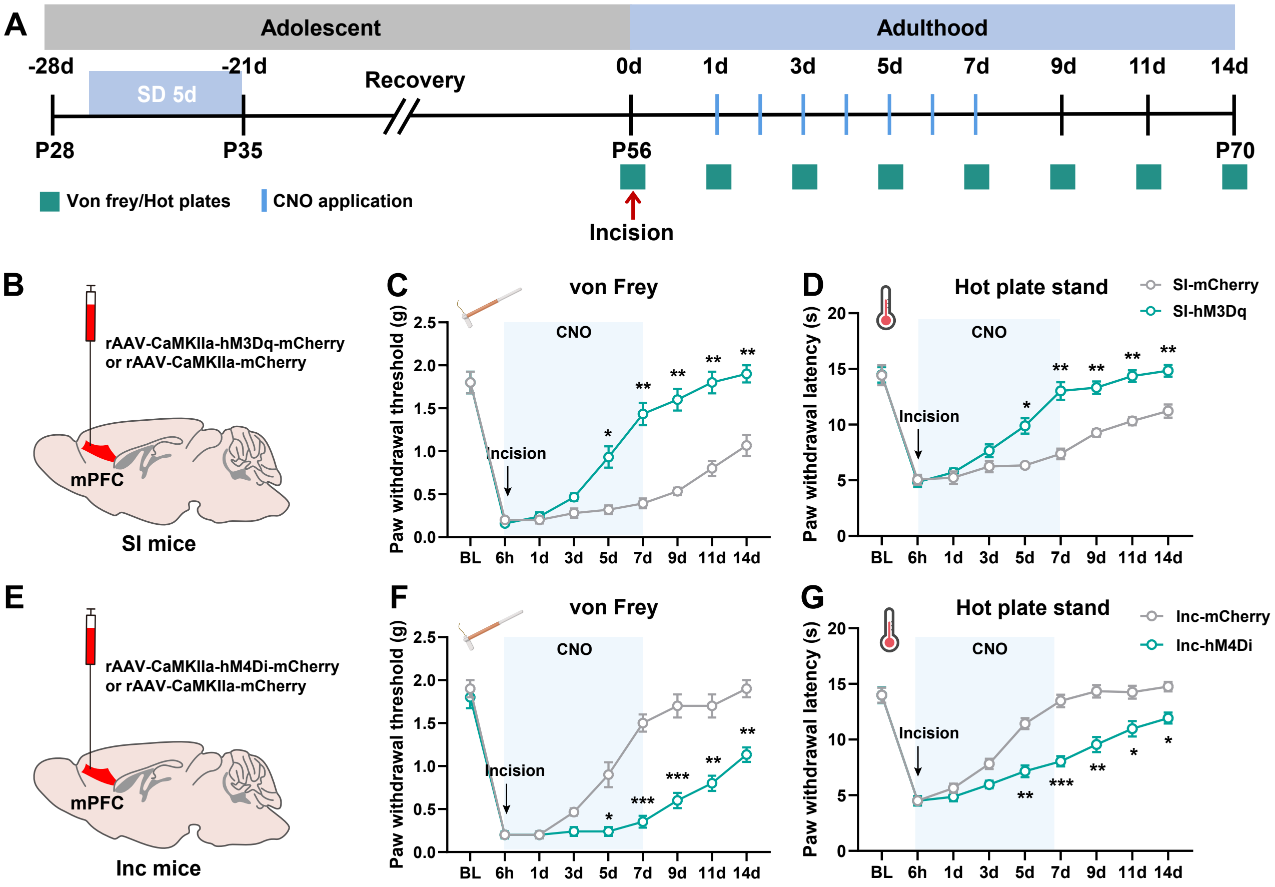

A
Adolescent
Adulthood
-28d
-21d
0d
1d
3d
5d
7d
9d
11d
14d
SD 5d
Recovery
P28
P35
P56
P70
Von frey/Hot plates
CNO application
Incision
B
rAAV-CaMKIIa-hM3Dq-mCherry
or rAAV-CaMKIIa-mCherry
mPFC
SI mice
C
von Frey
Paw withdrawal threshold (g)
CNO
Incision
BL 6h 1d 3d 5d 7d 9d 11d 14d
D
Hot plate stand
Paw withdrawal latency (s)
SI-mCherry
SI-hM3Dq
E
rAAV-CaMKIIa-hM4Di-mCherry
or rAAV-CaMKIIa-mCherry
mPFC
Inc mice
F
von Frey
G
Hot plate stand
Inc-mCherry
Inc-hM4Di
