# Supplementary material for: Targeting the Medial Prefrontal Cortex with Transcranial Focused Ultrasound Alleviates Adolescent Sleep-Deprivation-Induced Persistent Postsurgical Pain in Adulthood
Source: Research (Wash D C). 2026 Sep 23;9:1437. doi: 10.34133/research.1437 (PMC13598325; doi:10.34133/research.1437)
Supplement: Supplementary file 1 [file research.1437.f1.zip › FigureS6.pdf]

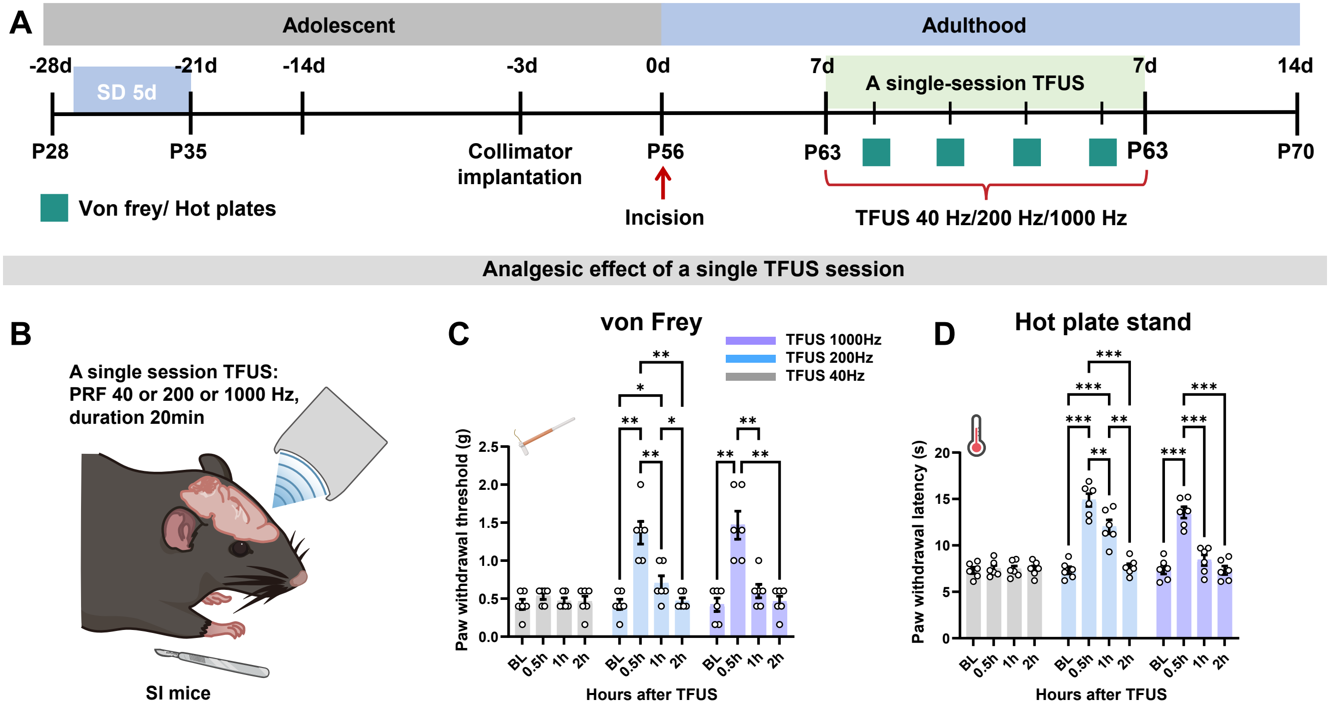
A
Adolescent
Adulthood
-28d
SD 5d
-21d
-14d
-3d
0d
7d
A single-session TFUS
7d
14d
P28
P35
Collimator implantation
P56
P63
P63
P70
Incision
Von frey/ Hot plates
TFUS 40 Hz/200 Hz/1000 Hz
Analgesic effect of a single TFUS session
B
A single session TFUS: PRF 40 or 200 or 1000 Hz, duration 20min
SI mice
C
von Frey
TFUS 1000Hz
TFUS 200Hz
TFUS 40Hz
Paw withdrawal threshold (g)
Hours after TFUS
BL
0.5h
1h
2h
D
Hot plate stand
Paw withdrawal latency (s)
Hours after TFUS
