# Supplementary material for: Targeting the Medial Prefrontal Cortex with Transcranial Focused Ultrasound Alleviates Adolescent Sleep-Deprivation-Induced Persistent Postsurgical Pain in Adulthood
Source: Research (Wash D C). 2026 Sep 23;9:1437. doi: 10.34133/research.1437 (PMC13598325; doi:10.34133/research.1437)
Supplement: Supplementary file 1 [file research.1437.f1.zip › FigureS7.pdf]

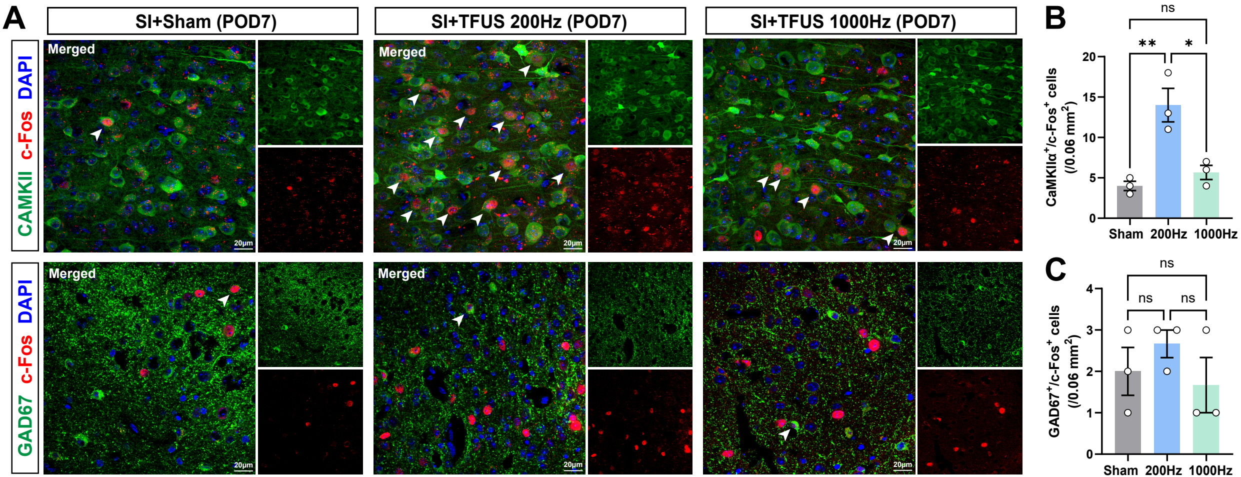

A
SI+Sham (POD7)
SI+TFUS 200Hz (POD7)
SI+TFUS 1000Hz (POD7)
CAMKII c-Fos DAPI
GAD67 c-Fos DAPI
Merged
20μm
B
CaMKIIα+/c-Fos+ cells (/0.06 mm2)
ns
**
*
Sham
200Hz
1000Hz
C
GAD67+/c-Fos+ cells (/0.06 mm2)
ns
ns
ns
Sham
200Hz
1000Hz
