# Supplementary material for: Targeting the Medial Prefrontal Cortex with Transcranial Focused Ultrasound Alleviates Adolescent Sleep-Deprivation-Induced Persistent Postsurgical Pain in Adulthood
Source: Research (Wash D C). 2026 Sep 23;9:1437. doi: 10.34133/research.1437 (PMC13598325; doi:10.34133/research.1437)
Supplement: Supplementary file 1 [file research.1437.f1.zip › FigureS8.pdf]

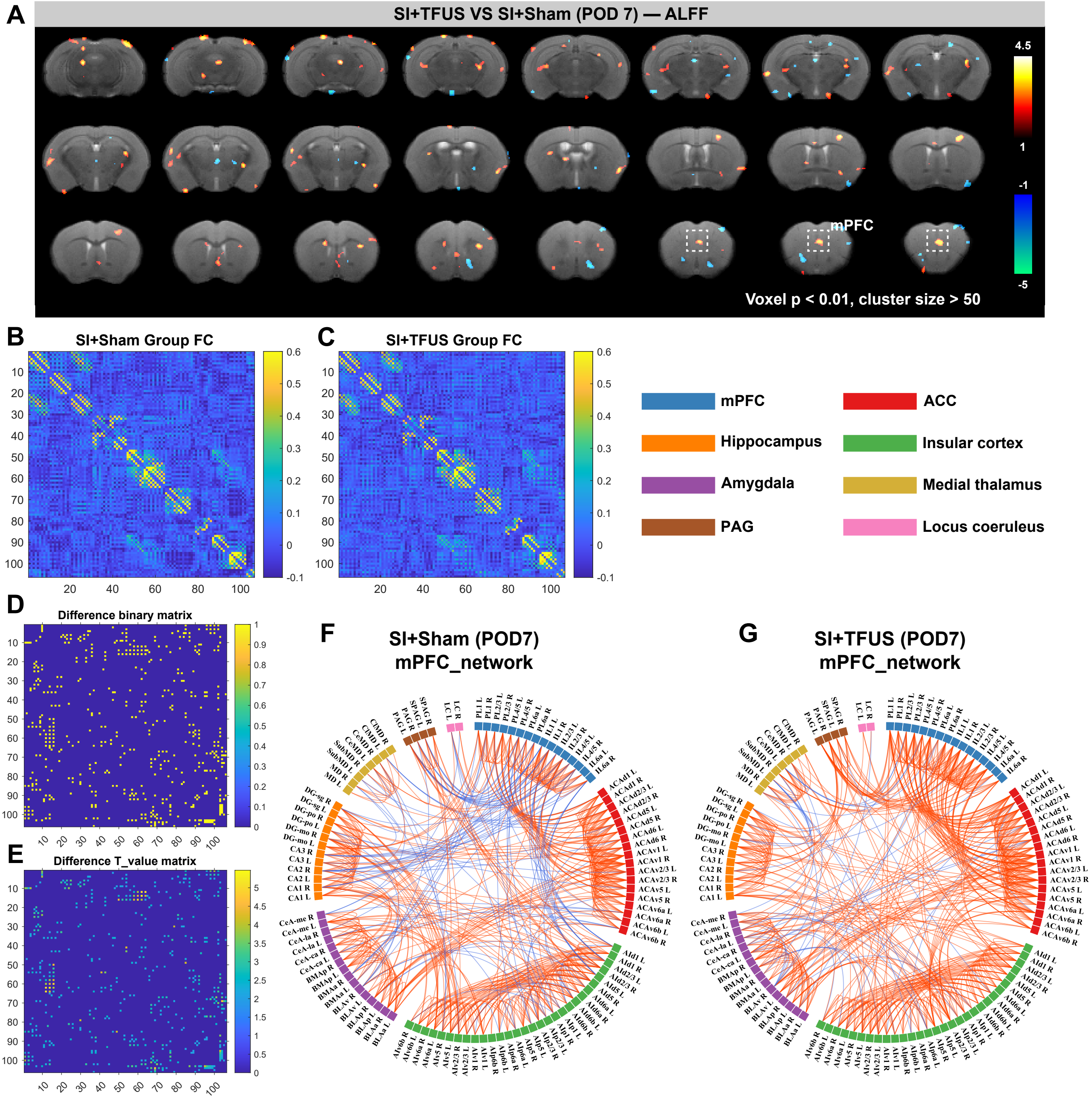
A
SI+TFUS VS SI+Sham (POD 7) — ALFF
mPFC
Voxel p < 0.01, cluster size > 50
B
SI+Sham Group FC
C
SI+TFUS Group FC
mPFC
ACC
Hippocampus
Insular cortex
Amygdala
Medial thalamus
PAG
Locus coeruleus
D
Difference binary matrix
E
Difference T_value matrix
F
SI+Sham (POD7)
mPFC_network
G
SI+TFUS (POD7)
mPFC_network
