# Supplementary material for: Targeting the Medial Prefrontal Cortex with Transcranial Focused Ultrasound Alleviates Adolescent Sleep-Deprivation-Induced Persistent Postsurgical Pain in Adulthood
Source: Research (Wash D C). 2026 Sep 23;9:1437. doi: 10.34133/research.1437 (PMC13598325; doi:10.34133/research.1437)

Synchronization of mPFC pyramidal neuronal activity and low gamma oscillation

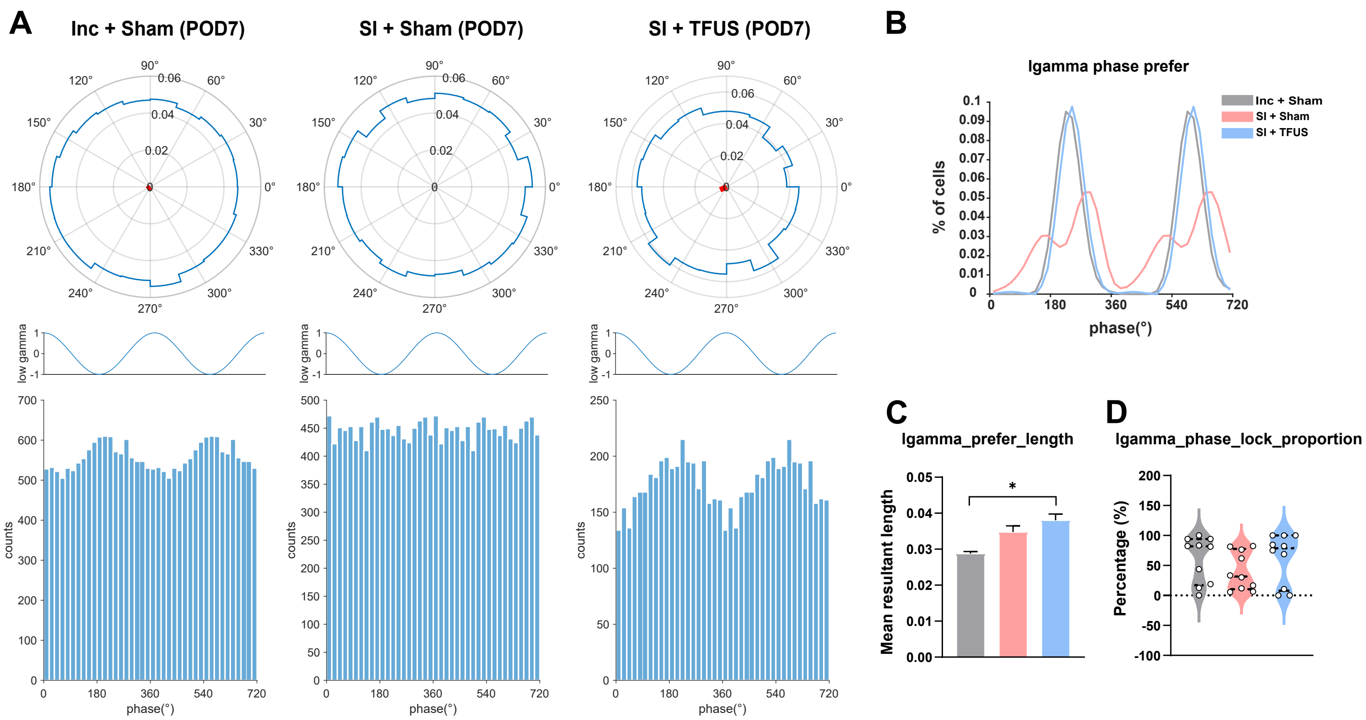

Supplement: Supplementary file 1 [file research.1437.f1.zip › FigureS9.pdf]
