# Supplementary material for: Targeting the Medial Prefrontal Cortex with Transcranial Focused Ultrasound Alleviates Adolescent Sleep-Deprivation-Induced Persistent Postsurgical Pain in Adulthood
Source: Research (Wash D C). 2026 Sep 23;9:1437. doi: 10.34133/research.1437 (PMC13598325; doi:10.34133/research.1437)
Supplement: Supplementary file 1 [file research.1437.f1.zip › Supplementary material-R2.docx]

**Supplementary material**

**Targeting the Medial Prefrontal Cortex with Transcranial Focused Ultrasound Alleviates Adolescent Sleep Deprivation-Induced Persistent Postsurgical Pain in Adulthood**

The supplementary material includes Materials and Methods, 15 Supplementary Figures and Figure Legends (Fig. S1 to S15), Supplementary Table 1-4.

**Materials and methods**

**Sleep deprivation protocol**

Adolescent mice underwent 5 days of SD using an automated treadmill system (XR-XS108, Shanghai Xin Ruan) based on a modified Bellesi protocol. To minimize stress, the SD protocol was initiated at the start of the dark phase (ZT12). From postnatal day (P) 30 to P35, mice were housed in acrylic cages with a continuously alternating rotating rod (7 rpm, 1.5 turns per direction) for 20 hours per day to prevent sleep while allowing free access to food and water. Control mice were housed identically but in static cages. Behavioral tests were conducted from P56 onward.

**Plantar incisional pain model**

The plantar incision procedure was performed based on an established murine model described previously. Under sterile conditions, mice were anesthetized with 1-3% isoflurane. A 7-mm longitudinal incision (starting 2 mm from the heel) was made through the skin and fascia of the right plantar surface using a number 11 scalpel. The underlying muscle was elevated and incised longitudinally, preserving its attachments. Following hemostasis, the wound was closed with a single 5-0 nylon suture and treated with erythromycin ointment. Mice recovered in a heated cage.

**Von Frey filament test**

Mice were individually housed in plexiglass chambers mounted on an elevated wire mesh platform. Animals underwent 30 min/day habituation sessions for 2 consecutive days to minimize environmental stress. Following a 30-min acclimatization period on the test day, mechanical withdrawal thresholds of the right hind paw plantar surface were quantified using ascending-force von Frey filaments (0.16g, 0.4g, 0.6g, 1.0g, 1.4g and 2.0 g; North Coast, USA). To prevent repetitive stimulation-induced escape behaviors or thermal injury, inter-stimulus intervals were maintained at ≥ 3 min.

**Hot plate test**

The hot plate test was performed as previously described. Mice were placed on a hot plate maintained at 52 ± 0.2 °C, and the latency to either hind paw licking or jumping was recorded. A 30 s cut-off was enforced to prevent tissue injury.

**Sleep recording surgery**

Mice were anesthetized with sodium pentobarbital (20 mg/kg, i.p.) and secured in a stereotaxic frame (RWD). Three stainless steel screw electrodes were stereotactically implanted for bilateral frontoparietal electrocorticography: two active electrodes and one cerebellar reference. For electromyography (EMG), Teflon-coated stain lesssteel wires were bilaterally inserted into cervical paraspinal muscles. All electrodes were anchored to the skull with dental cement. Seven days post-surgery, EEG electrodes were connected to recording systems, followed by a ≥ 2-day habituation period to minimize procedural stress.

**Electroencephalogram (EEG) and electromyography (EMG) recordings**

Following a 48-hour habituation period to the recording chamber and wireless headstage attachment, C57BL/6J mice were implanted with a lightweight (< 2.5 g) 4-channel wireless EEG system (Quanlan AR4-M). The system enabled continuous EEG/EMG monitoring with Bluetooth 5.0 transmission (500 Hz sampling rate, 0.5-35 Hz bandpass filtering) powered by a 35 mAh battery supporting ≥ 7-day recordings. Baseline 24-hour data were acquired under undisturbed conditions starting at dark cycle onset. Sleep stage scoring was performed using the AI-driven software Lunion Stage (https://www.luniondata.com/en/lunion_stage). This software utilizes a convolutional neural network to analyze EEG/EMG data in 4-second epochs, automatically classifying them as NREM sleep, REM sleep, or WAKE based on spectral and waveform characteristics. The automatically generated results were then subjected to manual review and correction. The scoring criteria were as follows: Wakefulness was identified by desynchronized EEG and high EMG activity; NREM sleep by synchronized, high-amplitude, low-frequency (0.1-4 Hz) EEG and low EMG activity; and REM sleep by a regular theta rhythm (4-8 Hz) with low EMG activity.

**Chemogenetic manipulation**

For targeted chemogenetic activation of glutamatergic neurons in the mPFC, the rAAV-CaMKIIα-hM3D(Gq)-mCherry-WPRE-pA (AAV2/9, 5.32×10^12^ vg/mL, BrainVTA) or rAAV-CaMKIIα-mCherry-WPRE-hGH pA (AAV2/9, 5.29×10^12^ vg/mL, BrainVTA), was stereotaxically delivered into the mPFC (150 nL per side). Following a three-week period to allow for viral expression, neuronal activation was induced by intraperitoneal administration of clozapine-N-oxide (Cat# C0832, Sigma-Aldrich) at a dose of 1 mg/kg. For sustained chemogenetic activation of mPFC^Glu^ neurons, CNO was administered once daily over seven consecutive days (POD 1-7). Behavioral assessments were conducted at least 30 minutes after CNO injection.

**Open field test (OFT)**

Mice were individually positioned in the center of a 40 × 40 × 40 cm open-field arena, digitally partitioned into 16 equal quadrants. Behavioral testing commenced with a 5-minute free exploration session under continuous video tracking (EthoVision XT v15.0, Noldus, Netherlands). Locomotor trajectories were analyzed for central zone entries (frequency) and dwell time (duration), with inter-trial apparatus decontamination using 75% ethanol (v/v) to eliminate residual olfactory cues.

**Elevated plus maze (EPM)**

The elevated plus maze (EPM) apparatus was constructed with four radially extending arms: two closed arms (30 × 6 × 20 cm; walls on three sides) and two open arms (30 × 6 cm; no walls), connected by a neutral central platform (6 × 6 cm). The maze was elevated 100 cm above ground level under controlled illumination (open arm illumination: 20 lux). Each trial began by positioning the mouse on the central platform facing a closed arm, allowing 5 min of unrestricted exploration. Behavioral parameters-including open arm duration time (%) and open arm entry frequency-were quantified using EthoVision XT software (Noldus). The area was cleaned between tests using 75% ethanol.

**Fiber photometry recording and data analysis**

For stereotaxic viral delivery and fiber optic implantation, mice were anesthetized with sodium pentobarbital (50 mg/kg, i.p.) and immobilized in a stereotaxic frame (RWD). Recombinant viruses were pressure-injected into the mPFC using a 1 μL Hamilton microsyringe with a 28-gauge injector (Plastics One). One week post-injection, low-auto fluorescence fiber optic cannulas (RWD Life Science, 200um core, 0.39 NA, OD 1.25 mm) were implanted above the mPFC and secured with dental cement. For combined TFUS-fiber photometry experiments, a custom collimator was affixed to the skull above mPFC using cyanoacrylate adhesive (3M™), enabling angled fiber insertion along the collimator channel to target mPFC, followed by permanent cementation of both components. All coordinates are relative to bregma. Calcium-dependent fluorescence signals were acquired using a multichannel fiber photometry system (R820, RWD Life Science). GCaMP6s was excited with a 470 nm laser, and the resulting fluorescence was measured as an indicator of neuronal activity. A 410 nm laser was used concurrently as an isosbestic control for the signal.

**In vivo Electrophysiology Recording**

To monitor neuronal signals, the microdrive connectors were interfaced with preamplifiers connected via extended cables to the NeuroXess CereCube system (Naohu Technology, China). A helium-filled mylar balloon was tethered to the cable assembly to counteract its weight, allowing the mouse to move freely. Extracellular signals were filtered through the preamplifiers to separate spike activity from local field potentials (LFP). Spike signals were sampled at 40 kHz and band-pass filtered online at 400-7000 Hz, while LFP signals were sampled at 2 kHz and low-pass filtered at 300 Hz. All data, including spike waveforms, timestamps, and LFP signals, were saved in *.mex data files.

**Spike sorting**

Individual neurons were sorted by clustering methods in Offline Sorter software (Plexon), performed manually in a three-dimensional feature space.

**Spectral Analysis**

Local field potential (LFP) signals were analyzed to extract spectral features for intergroup comparisons. Spectral processing was performed using custom MATLAB scripts. The LFP signals, downsampled to a sampling rate of 1 kHz, were segmented within a predefined time window of 0-100 s for analysis. First, we applied a zero-phase 2nd-order Butterworth notch filter (49-51 Hz) to the raw data to suppress the main 50 Hz interference. Next, to mitigate minor spectral leakage introduced by the filter, we performed a piecewise cubic Hermite interpolating polynomial (pchip) interpolation between 48 Hz and 52 Hz. Power spectral density (PSD) was estimated using Welch’s method with a Hamming window of 2000 points (2 s duration), 50% overlap, and an FFT length of 8192. To account for inter-individual baseline variations, the obtained PSD was normalized by the total spectral power across the entire analyzed frequency range (0.5-100 Hz) to yield the relative PSD.

Time-frequency analysis was conducted using the short-time Fourier transform (STFT) across a frequency range of 0.1-100 Hz for spectrogram visualization.

Spectral power was quantified within six frequency bands: delta (0.5-4 Hz), theta (4-8 Hz), alpha (8-12 Hz), beta (12-30 Hz), low gamma (30-60 Hz), and high gamma (60-100 Hz). For each band, the relative power was calculated. For each band, the relative PSD was calculated, extracted for each recording, and used for subsequent statistical comparisons across experimental groups.

**Phase‑Locking Analysis**

To examine the phase relationship between single-unit spiking activity and local field potential (LFP) oscillations, a phase-locking analysis was performed. LFP signals recorded at 2 kHz were first band-pass filtered within specific frequency bands-low gamma (30-60 Hz), high gamma (60-100 Hz), and broadband gamma (30-100 Hz)-using a second-order Butterworth filter with zero-phase distortion via the filtfilt function. The instantaneous phase of the filtered LFP was then extracted using the Hilbert transform, producing a continuous phase time series ranging from 0 to 2π, with the oscillation peak defined at 0 or 2π.

Spike times from sorted single units were aligned to the LFP time series. For each unit, spike-triggered LFP phases were calculated by sampling the instantaneous phase at the time of each spike (rounded to the nearest sample index). Units with fewer than 40 spikes during the analysis window (0-100 s) were excluded from further analysis to ensure statistical reliability.

To assess the significance of phase locking, the Rayleigh test for circular uniformity was applied to the distribution of spike phases. Neurons were considered significantly phase-locked if the resulting p-value was less than 0.05. The strength of phase locking was quantified by the mean resultant length (MRL), computed as the magnitude of the mean vector of the circular distribution of spike phases. MRL values range from 0 (random distribution) to 1 (perfect phase locking). Additionally, preferred phase angles were derived from the circular mean of significantly locked units. Phase distributions were visualized using polar histograms (20 bins over 360°) and conventional linear histograms. The proportion of phase-locked neurons in each frequency band was calculated per animal for group-level comparisons.

**Single-nucleus RNA-sequencing**

Single-cell capture and library preparation were performed using the 10x Genomics Chromium Next GEM Single Cell 3’ Kit v3 following manufacturer protocols. Libraries underwent paired-end sequencing (150 bp) on an Illumina NovaSeq 6000 platform (LC-Bio, China) with a minimum depth of 20,000 reads/cell. Raw sequencing data were demultiplexed using bcl2fastq (Illumina), followed by cell barcode processing and gene counting via Cell Ranger 4.0.0 aligned to the GRCm38 reference genome (Ensembl v96). Initial capture yielded 97467 cells (32800 I+Sham vs 30635 SI+Sham vs 34032 SI+TFUS), with stringent quality control retaining 77477 cells (26729 I+Sham vs 24845 SI+Sham vs 25903 SI+TFUS) based on thresholds: 500-Inf genes/cell and < 2.5% mitochondrial gene content. Data normalization employed the Log-Normalize method (Seurat 4.0.6) to mitigate sequencing depth variability. After identifying highly variable genes (Find Variable Features), we integrated I+Sham, SI+Sham and SI+TFUS datasets (Find Integration Anchors/Integrate Data) to eliminate batch effects. Scaled data underwent PCA-driven clustering (resolution=1) and UMAP visualization, revealing 41 distinct clusters subsequently annotated into 8 cell types using canonical marker genes: excitatory neurons (Slc17a7, Neurod6, Grin1, Grin2a), inhibitory neurons (Gad1, Gad2, Slc32al), microglia (Csf1r, Itgb5, Cx3cr1, Ctss, Tmem119), astrocytes (Fgfr3, Aqp4, Atp13a4, Aldoc, Mlc1, Gja1), oligodendrocytes (Plp1, Mog, Mbp), oligodendrocyte precursor cells (Gpr17, Pdgfra, Vcan), endothelia (Flt1, Cldn5, Pecam1, Slc2a1), and Pericytes (Vtn). Differential gene expression (DEG) analysis was conducted with parameters min.pct = 0.25 and logFC.threshold=1.

**fMRI data acquisition**

Functional MRI experiments were conducted at three critical developmental timepoints: on the day following adolescent sleep deprivation (P35), adulthood following adolescent sleep deprivation (P56), and 7 days post-plantar incision in adulthood (P63). Von Frey mechanical allodynia assessments preceded each fMRI session to establish baseline nociceptive thresholds. Animal preparation followed our established protocol using a 9.4 T Bruker BioSpec 70/20 USR system with a 20 cm horizontal bore. Mice were initially anesthetized with 3-4% isoflurane and maintained at 1-1.5% throughout imaging, with body temperature regulated via heating pad. Precise stereotaxic positioning was achieved using ear and bite bars to ensure standardized brain alignment. Functional data acquisition employed a multi-slice gradient-echo EPI sequence (TR = 1000 ms, TE = 14 ms, flip angle = 45°, field of view = 20×20 mm², resolution = 0.31×0.31×1 mm³, 20 slices). High-resolution anatomical references were obtained via T2-weighted RARE sequences (TR = 5000 ms, TE = 12 ms, RARE factor = 8, matrix = 256×256, resolution = 0.08×0.08 mm²) matching fMRI geometry. Our analysis incorporated two complementary methods: ALFF and ReHo. ALFF reflects the intensity of spontaneous neural activity in each voxel from an energy metabolism perspective. In contrast, ReHo is a metric that assesses the temporal synchrony and coherence of neural activity within a local brain region. As ALFF and ReHo provide distinct information regarding different types of neuronal activity, they serve as complementary approaches for investigating whole-brain alterations.

**In vivo knockdown of TRPC5**

To achieve TRPC5 knockdown (TRPC5-KD), AAV vectors encoding mCaMKIIa-MasterRNAi155 (Trpc5) (AAV2/9-WY4840, Taitool) or a scrambled control AAV (S0853-9-H20, Taitool) were stereotaxically microinjected into bilateral mPFC (300 nL/hemisphere; 10⁶ infectious units/mL). Three weeks post-injection, mice received mPFC-targeted TFUS stimulation as detailed in prior methodology. TRPC5 protein levels in mPFC lysates were quantified via immunoblotting using anti-TRPC5 antibodies and normalized to β-actin, with comparisons between TRPC5-KD and scrambled shRNA-injected controls.

**In vivo overexpression of Homer1**

To achieve Homer1 overexpression (Homer1^Ove^), AAV vectors encoding mCaMKIIa-Homer1-3×FLAG (AAV2/9-WY6321, Taitool) or a scrambled control AAV were stereotaxically microinjected into bilateral mPFC (300 nL/hemisphere; 10⁶ infectious units/mL). Three weeks post-injection, SD mice underwent plantar incision, followed by behavioral testing and subsequent experiments.

**Reagents**

For mechanosensitive ion channel inhibition, we mixed GsMTx4 (MedChem Express, HY-P1410) with artificial cerebrospinal fluid (aCSF) and injected it (1 μM, 500 nl) into the mPFC 30 min before TFUS application. Similarly, to selectively inhibit TRPC5 activity, we locally injected AC1903 (MedChem Express, HY-122051) (50 μM, 500 nl) into the mPFC via an implanted cannula 30 min prior to TFUS.

**Golgi staining**

Golgi staining was conducted on mPFC samples according to the instructions provided in the FD Rapid GolgiStain™ Kit (FD NeuroTechnologies, PK401). Briefly, following anesthesia with isoflurane, brain tissues were quickly extracted and gently washed in physiological saline. They were then immersed in a freshly prepared combination of Solution A and Solution B and kept in darkness at room temperature for 14 days. The immersion solution was changed 24 hours after the initial incubation. Thereafter, the tissue was placed into Solution C and stored in the dark for an additional week, with the solution also replaced after the first 24 hours. Using a freezing microtome (Leica CM3050S, Germany), 100 μm thick sections were obtained and allowed to dry under dark conditions. These sections were subsequently rinsed twice with ddH₂O water, followed by incubation for 10 min in a 50 mL mixture of Solution D, Solution E, and ddH₂O water mixed at a ratio of 1:1:2. After two further rinses with ddH₂O water, the sections were dehydrated through a graded ethanol series, cleared in xylene, and mounted with Permount. Neuronal dendritic morphology in the mPFC was visualized under a confocal microscope (Leica Stellaris5, Germany) using 20× and 100× objectives. Dendritic spine analysis was performed with the Cell Counter plugin in Image J software.

**Western blot analysis**

Bilateral mPFC tissue was dissected from 500-μm-thick coronal sections prepared using a mouse brain matrix. Tissue samples were mechanically homogenized in ice-cold RIPA lysis buffer (supplemented with phosphatase/protease inhibitors) using a 29-gauge needle, followed by 20-min lysis on ice. Lysates were clarified by centrifugation (12,000 × g, 15 min, 4°C). Protein extracts (20 μg per lane) were separated by SDS-PAGE and transferred to Immobilon-P PVDF membranes (Millipore Sigma) for immunoblotting. After blocking with 5% nonfat milk, the membranes were incubated with primary antibodies, including GAPDH (1:1000, Proteintech, 60004-1-Ig), TRPC5 (1:1000, alomone, ACC-020), Homer1 (1:1000, Abcam, ab184955), Homer1b/c (1:1000, Synaptic Systems, 160023), GluA1 (1:1000, Abcam, ab31232), GluA2 (1:1000, Abcam, ab206293), NR1 (1:1000, Invitrogen, 32-0500), NR2A (1:1000, Proteintech, 28525-1-AP), NR2B (1:1000, Proteintech, 21920-1-AP) and PSD95 (1:1000, Proteintech, 20665-1-AP) at 4℃ overnight and peroxidase-labeled secondary antibody (1:5000, Thermo Scientific) at room temperature for 90 min. Protein bands were visualized by chemiluminescence and quantified using ImageJ software.

**Immunohistochemistry and imaging**

Brains were harvested 30 min after behavioral testing for c-Fos immunofluorescence staining. Mice were deeply anesthetized via intraperitoneal (i.p.) injection of sodium pentobarbital (50 mg/kg) and transcardially perfused with ice-cold physiological saline (0.9% NaCl) followed by 4% PFA in PBS. Brains were dissected, post-fixed in 4% PFA/PBS at 4°C for 24 h, then cryoprotected in 30% sucrose/PBS until tissue saturation. Coronally oriented sections (20 μm) were obtained using a cryostat (Leica, Germany, CM3050S). For immunofluorescence, sections underwent sequential processing: (1) 1 h blocking in 0.3% Triton X-100/10% donkey serum/PBS at RT; (2) 24 h primary antibody incubation (4°C) in blocking solution; (3) PBS washes; (4) species-matched secondary antibody incubation (2 h, RT). Finally, brain sections were PBS-rinsed and coverslipped with antifade mounting medium. The following primary or secondary antibodies were used in the current study at the indicated dilutions: rabbit anti-cFos (1:500, CST, 2250S), Guinea piganti-GAD67 (1:200, Oasis, OB-PGP074-01), donkey anti-rabbit AlexaFluor 594 (1:1000, Invitrogen, A-21207) and donkey anti-rabbit AlexaFluor 488 (1:1000, Invitrogen, A-21206). The stained sections were visualized using either a confocal microscope (Stellaris5, Leica, Germany) or a whole-slide scanner (VS120, Olympus, Japan).

**Supplementary Figures and Figure Legends**

**Supplementary Figure 1. Establishment of an adolescent SD model paradigm in mice.**

**(A-B)** Adolescent SD protocol and timeline of experimental procedures. **(C)** Examples of relative EEG power and EMG/ACC traces in adolescent mice (P30-35) over a 24-hr light-dark cycle of Con or SD group at 20:00. **(D-F)** Time course changes in wakefulness, NREM sleep, and REM sleep (n = 5 mice per group; Two-way ANOVA, Wake: *F*_1,8_ = 33.82, *P* = 0.0004; NREM: *F*_1,8_ = 22.02, *P* = 0.0016; REM: *F*_1,8_ = 43.00, *P* = 0.0002). **(G-I)** Total amount of time, bout number and average bout length of Wake, NREM and REM states. Unpaired t-test within each state, **(G)** Wake, *t*_8_ = 5.8, *P* = 0.0004; NREM, *t*_8_ = 4.680, *P* = 0.0016; REM, *t*_8_ = 6.557, *P* = 0.0002; **(H)** Wake, *t*_8_ = 4.372, *P* = 0.0024; NREM, *t*_8_ = 4.612, *P* = 0.0017; REM, *t*_8_ = 12.86, *P* < 0.0001; **(I)** Wake, *t*_8_ = 3.808, *P* = 0.0052; NREM, *t*_8_ = 9.834, *P* < 0.0001. **(J-L)** EEG power spectrum of WAKE, NREM and REM sleep was altered after 5 days of SD (Unpaired t-test). Con, control. SD, sleep deprivation. NREM, Non-rapid eye movement. REM, rapid eye movement. PSD, power spectral density. Data are presented as means ± SEM. **P* < 0.05; ***P* < 0.01; ****P* < 0.001.

**Supplementary Figure 2. Normalization of pain thresholds and sleep architecture in adulthood after adolescent SD**

**(A)** Timeline of experimental procedures. **(B-C)** Schematic diagram and statistical graph of the von Frey test and hot plate test at P56 (n = 6, Unpaired t-test). **(D)** Examples of relative EEG power and EMG/ACC traces in adult mice (P56) over 24 h of Con or SD group at 20:00. **(E-G)** Time course changes in WAKE, NREM, and REM (n = 5; Two-way ANOVA, Wake: *F*_1,8_ = 0.2859, *P* = 0.6074; NREM: *F*_1,8_ = 0.3070, *P* = 0.5947; REM: *F*_1,8_ = 0.0146, *P* = 0.9070). **(H-J)** Total amount of time, bout number and average bout length of Wake, NREM and REM states. Unpaired t-test within each state, **(H)** Wake, t_8_ = 0.5423, *P* = 0.6024; NREM, t_8_ = 0.5546, *P* = 0.5943; REM, t_8_ = 0.1294, *P* = 0.9002; **(I)** Wake, t_8_ = 0.5341, *P* = 0.6078; NREM, t_8_ = 0.6531, *P* = 0.5320; REM, t_8_ = 0.4487, *P* = 0.6655; **(J)** Wake, t_8_ = 0.1932, *P* = 0.8516; NREM, t_8_ = 0.6799, *P* = 0.5158; REM, t_8_ = 0.7644, *P* = 0.4666. **(K-M)** EEG power spectrum of WAKE, NREM and REM sleep was not altered of SD mice compared to Con mice (Unpaired t-test). Con, control. SD, sleep deprivation. NREM, Non-rapid eye movement. REM, rapid eye movement. PSD, power spectral density. Data are presented as means ± SEM.

**Supplementary Figure 3. Adolescent sleep deprivation induces persistent post-surgical pain in adult female mice**

**(A)** Schematic of the adolescent SD and adult incision model in female mice. **(B-C)** Time course assessment of mechanical and thermal nociceptive thresholds in the right hindpaw of female mice (n = 6; Two-way repeated-measures ANOVA with post hoc comparison). Con, control. SD, sleep deprivation. Inc, incision. SI, sleep deprivation combined with incision. Data are presented as means ± SEM. SI vs Inc, **P* < 0.05; ***P* < 0.01; ****P* < 0.001.

**Supplementary Figure 4. Bidirectional chemogenetic modulation of mPFCGlu neurons alters postoperative pain behaviors**

**(A)** Timeline of experimental procedures. **(B, E)** Schematic representation of the virus injections for chemogenetic activation and inhibition, respectively. **(C-D)** Time course assessment of nociceptive thresholds in the right hindpaw following chemogenetic activation (hM3Dq) in SI mice (n = 6 mice per group; Two-way repeated-measures ANOVA with post hoc comparison; von Frey, *F*_1,10_ = 43.95, *P* < 0.0001; Hot plate, *F*_1,10_ = 46.88, *P* < 0.0001). **(F-G)** Time course assessment of nociceptive thresholds in the right hindpaw following chemogenetic inhibition (hM4Di) in Inc mice (n = 6 mice per group; Two-way repeated-measures ANOVA with post hoc comparison; von Frey, *F*_1,10_ = 90.43, *P* < 0.0001; Hot plate, *F*_1,10_ = 30.79, *P* = 0.0002). Inc, incision. SI, sleep deprivation combined with incision. BL, baseline. CNO, Clozapine N-Oxide. Data are presented as means ± SEM. **P* < 0.05; ***P* < 0.01; ****P* < 0.001.

**Supplementary Figure 5. The c-Fos expression in mice's different brain regions**

**(A-D)** Representative images of c-Fos immunohistochemistry in cortical regions adjacent to the mPFC of TFUS-treated mice. **(E-F)** Representative pictures of c-Fos immunohistochemistry in the auditory cortex of TFUS-treated mice (left) and Sham-treated mice (right). mPFC, medial prefrontal cortex. TFUS, transcranial focused ultrasound.

**Supplementary Figure 6. Analgesic effects of a single TFUS session**

**(A)** Similar to Fig. 1A, but combined with a single-session of TFUS stimulation. **(B)** Schematic of a single session TFUS protocol in a freely moving mouse. **(C-D)** Assessment of nociceptive thresholds at multiple time points following a single TFUS session (n = 6 mice per group; Two-way repeated-measures ANOVA with post hoc comparison; von Frey, *F*_1.747,26.20_ = 96.64, *P* < 0.0001; Hot plate, *F*_2.064,30.97_ = 315.9, *P* < 0.0001). BL, baseline. SI, sleep deprivation combined with incision. TFUS, transcranial focused ultrasound. PRF, pulse repetition frequency. Data are presented as means ± SEM. **P* < 0.05; ***P* < 0.01; ****P* < 0.001.

**Supplementary Figure 7. 200 Hz PRF TFUS selectively restores the activity of mPFC glutamatergic neurons in SI mice**

**(A-C)** Representative images and quantification of c-Fos/CaMKIIα and c-Fos/GAD67 double immunofluorescence staining in the mPFC (n = 3 mice per group; One-way ANOVA with post hoc comparison). Scale bar, 20 μm. mPFC, medial prefrontal cortex. SI, sleep deprivation combined with incision. TFUS, transcranial focused ultrasound. POD 7, postoperative day 7. PRF, pulse repetition frequency. Data are presented as means ± SEM. **P* < 0.05; ***P* < 0.01; ****P* < 0.001.

**Supplementary Figure 8. TFUS restores local mPFC activity and enhances pain-related network connectivity**

**(A)** Voxel-wise analysis showing ALFF alterations between the SI+Sham and SI+TFUS groups on POD 7 (n = 8 per group). **(B-C)** Group-averaged FC matrices for the pain-related network. Numbers correspond to the 106 subregions detailed in Supplementary Table 4. Color bars indicate correlation coefficients. **(D)** Difference adjacency matrix (*P* = 0.0192) indicating enhanced network connectivity in the SI+TFUS group compared to the SI+Sham group. **(E)** Statistical t-value matrix. Data were analyzed using paired t-tests and network-based statistics (NBS) with 5000 permutations (significance defined as P < 0.05). **(F-G)** Connectograms illustrating the top 10% strongest functional connections in the SI+Sham and SI+TFUS groups. Edge thickness represents connection strength. SI, sleep deprivation combined with incision. TFUS, transcranial focused ultrasound. POD 7, postoperative day 7. ALFF, amplitude of low-frequency fluctuation. FC, functional connectivity. mPFC, medial prefrontal cortex. ACC, anterior cingulate cortex. PAG, periaqueductal gray. LC, locus coeruleus.

**Supplementary Figure 9. Effect of TFUS on phase-locking of mPFC neurons to low gamma oscillations**

**(A)** Representative phase-locking plots in the mPFC relative to local low gamma oscillations across the Inc+Sham, SI+Sham, and SI+TFUS groups. **(B)** Low gamma phase preference plot. **(C)** The strength of mPFC neuron phase-locking to local low gamma oscillations showed no significant difference between the groups (Kruskal-Wallis test). **(D)** The proportion of phase-locking mPFC neurons showed no significant difference among groups for low gamma band (Kruskal-Wallis test). mPFC, medial prefrontal cortex. Inc, incision. SI, sleep deprivation combined with incision. TFUS, transcranial focused ultrasound. POD 7, postoperative day 7. Data are presented as means ± SEM. **P* < 0.05.

**Supplementary Figure 10. snRNA-seq cell type analysis.**

**(A-B)** Cell-type distribution by groups. **(C, F)** Comparative analysis of Homer1 expression by volcano plot across experimental groups (SI+Sham vs I+Sham; SI+TFUS vs SI+Sham) in excitatory neurons. **(D-E)** Enrichment of KEGG pathways and GO terms from the SI+Sham vs I+Sham DEGs. **(G-H)** Enrichment of KEGG pathways and GO terms from the SI+TFUS vs SI+Sham DEGs. Inc, incision. SI, sleep deprivation combined with incision. POD 7, postoperative day 7. TFUS, transcranial focused ultrasound. DEGs, differentially expressed genes. KEGG, kyoto encyclopedia of genes and genomes. GO, gene ontology. Data are presented as means ± SEM.

**Supplementary Figure 11. TRPC5 mediates TFUS-induced calcium transients and selectively regulates the activity of mPFC glutamatergic neurons**

**(A-B)** Heatmaps and representative average ΔF/F traces of fiber photometry recordings in vehicle- or AC1903-treated mice following a single-session TFUS stimulation (n = 4 mice per group, with 3 trials conducted per mouse). **(C)** Statistics of peak amplitudes (Unpaired t-test) between changes across mice. **(D-F)** Representative images and quantification of c-Fos/CaMKIIα and c-Fos/GAD67 double immunofluorescence staining in the mPFC of TRPC5^Veh^ and TRPC5^KD^ mice (n = 3 mice per group; Unpaired t-test). Scale bar, 20 μm. mPFC, medial prefrontal cortex. SI, sleep deprivation combined with incision. TFUS, transcranial focused ultrasound. POD 7, postoperative day 7. TRPC5, transient receptor potential channel 5. TRPC5^KD^, TRPC5 knockdown. TRPC5^Veh^, TRPC5 vehicle control. Data are presented as means ± SEM. **P* < 0.05; ***P* < 0.01; ****P* < 0.001.

**Supplementary Figure 12. Synaptic enrichment of Homer1 and AMPAR subunits in the mPFC**

**(A-E)** Representative western blots and quantitative data of Homer1, GluA1, and GluA2 levels (normalized to PSD95), and PSD95 levels (normalized to GAPDH), in synaptosomal fractions from the mPFC across Con, SD, Inc, and SI mice (POD 7, n = 3, One-way ANOVA with post hoc comparison). **(F-J)** Representative western blots and quantitative data of Homer1, GluA1, and GluA2 levels (normalized to PSD95), and PSD95 levels (normalized to GAPDH), in synaptosomal fractions from the mPFC across SI+Sham, SI+TFUS, SI+TFUS+TRPC5^Veh^, and SI+TFUS+TRPC5^KD^ mice (POD 7, n = 3, One-way ANOVA with post hoc comparison). Con, control. SD, sleep deprivation. Inc, incision. SI, sleep deprivation combined with incision. TFUS, transcranial focused ultrasound. POD 7, postoperative day 7. TRPC5, transient receptor potential channel 5. TRPC5^KD^, TRPC5 knockdown. TRPC5^Veh^, TRPC5 vehicle control. Data are presented as means ± SEM. **P* < 0.05; ***P* < 0.01; ****P* < 0.001.

**Supplementary Figure 13. Homer1 overexpression in the mPFC rescues dendritic spine deficits and alleviates PPSP in SI mice.**

**(A-B)** Time course assessment of mechanical and thermal nociceptive thresholds following Homer1 overexpression (n = 6; Two-way repeated-measures ANOVA with post hoc comparison). **(C-D)** Representative Golgi staining images and quantification of dendritic spine density in mPFC pyramidal neurons (n = 20 neurons from 4 mice per group, Unpaired t-test). BL, baseline. SI, sleep deprivation combined with incision. Homer1^Ove^, Homer1 overexpression. Homer1^Veh^, Homer1 vehicle control. Data are presented as means ± SEM. **P* < 0.05; ***P* < 0.01; ****P* < 0.001.

**Supplementary Figure 14. Pharmacological inhibition of mechanosensitive cation channels blocks TFUS-induced analgesia.**

**(A-B)** Time course assessment of mechanical and thermal nociceptive thresholds following intra-mPFC injection of GsMTx4 prior to TFUS treatment in SI mice (n = 6 mice per group; two-way repeated-measures ANOVA with post hoc comparison). BL, baseline. SI, sleep deprivation combined with incision. TFUS, transcranial focused ultrasound. Data are presented as means ± SEM. **P* < 0.05; ***P* < 0.01; ****P* < 0.001.

**Supplementary Figure 15. Adolescent SD predisposes mice to adult PPSP, which can be therapeutically alleviated by mPFC-targeted TFUS.**

Adolescent SD (P30-P35) induces mPFC^Glu^ hypoactivity, predisposing mice to adult PPSP following hindpaw incision. By POD 7 (P63), SI mice display aberrant gamma oscillations, disrupted spike-gamma phase locking, and impaired synaptic plasticity linked to downregulated Homer1 and AMPAR expression. Conversely, targeted TFUS (200 Hz PRF) to the mPFC activates mechanosensitive TRPC5 channels to promote Homer1-dependent synaptic plasticity. This cascade reverses mPFC^Glu^ hypoactivity and normalizes local neural rhythms, thereby alleviating pain behaviors. Together, these findings identify mPFC^Glu^ neurons as a critical mediator for PPSP vulnerability induced by early-life sleep disturbances, and highlight TFUS as a potential noninvasive intervention for PPSP.

**Supplementary Table 1. Administered TFUS conditions with a constant DC of 10%.**

| **TFUS conditions^a^** | **PRF (Hz)** | **TBD (ms)** | **Cycles per pulse** |
| --- | --- | --- | --- |
| PRF 40Hz DC 10% | 40 | 2.5 | 5000 |
| PRF 200Hz DC 10% | 200 | 0.5 | 1000 |
| PRF 1000Hz DC 10% | 1000 | 0.1 | 200 |

^a^Except for the PRF and TBD, all of the listed TFUS conditions keep the following parameters constant, i.e., f_0_ = 2 MHz, UD = 1 s, ISoI = 8 s, I_SPTA_ = 84.38 mW/cm^2^, and I_SPPA_ = 6.75 W/cm^2^. TFUS, transcranial focused ultrasound. f_0_, fundamental frequency. PRF, pulse repetition frequency. DC, duty cycle. TBD, tone-burst duration. UD, ultrasound duration. ISoI, inter-sonication interval.

**Supplementary Table 2. Brain regions with significant ALFF alterations between the SI and Inc groups**

| ROI | Ke |
| --- | --- |
| Cluster1 | 49 |
| Insular Cortex_left | 13 |
| Cluster2 | 67 |
| Cluster3 | 46 |
| Hypothalamus_left | 1 |
| Cluster4 | 23 |
| Piriform Cortex_left | 23 |
| Cluster5 | 43 |
| Amygdala_left | 22 |
| Piriform Cortex_left | 31 |
| Cluster6 | 48 |
| Ectorhinal Cortex_left | 31 |
| Hippocampus_left | 6 |
| Subiculum_left | 35 |
| Temporal Cortex_left | 4 |
| Cluster7 | 28 |
| Caudate Putamen_right | 13 |
| Insular Cortex_right | 20 |
| Cluster8 | 485 |
| Caudate Putamen_left | 5 |
| Hippocampus_left | 75 |
| Thalamus_left | 105 |
| Cluster9 | 44 |
| DG_left | 10 |
| Hippocampus_left | 34 |
| Subiculum_left | 3 |
| Cluster10 | 57 |
| Caudate Putamen_right | 57 |
| Cluster11 | 31 |
| Caudate Putamen_left | 2 |
| Thalamus_left | 11 |
| Cluster12 | 56 |
| Cluster13 | 192 |
| Cluster14 | 78 |
| PFC_left | 43 |
| Septal_left | 2 |
| Cluster15 | 53 |
| Cingulate Cortex_left | 8 |
| M1_left | 20 |
| M2_left | 16 |
| Motor Cortex_left | 48 |

**Supplementary Table 3. Brain regions with significant ReHo alterations between the SI and Inc groups**

| ROI | Ke |
| --- | --- |
| Cluster1 | 41 |
| Piriform Cortex_left | 41 |
| Cluster2 | 125 |
| Accumbens Nucleus_left | 65 |
| Caudate Putamen_left | 53 |
| PFC_left | 47 |
| Piriform Cortex_left | 4 |
| Septal_left | 49 |
| Cluster3 | 23 |
| Hypothalamus_left | 9 |
| Substantia Nigra_left | 11 |
| Cluster4 | 74 |
| DG_left | 30 |
| Hippocampus_left | 66 |
| Cluster5 | 27 |
| Insular Cortex_right | 27 |
| Somatosensory Cortex_right | 3 |
| Cluster6 | 45 |
| DG_right | 11 |
| Hippocampus_right | 11 |
| Subiculum_right | 35 |
| Cluster7 | 31 |
| Thalamus_left | 23 |
| Cluster8 | 25 |
| Thalamus_left | 22 |
| Cluster9 | 25 |
| Hippocampus_right | 25 |
| Cluster10 | 20 |
| DG_right | 2 |
| Hippocampus_right | 20 |
| Cluster11 | 157 |
| DG_left | 12 |
| DG_right | 1 |
| Habenula Nucleus_left | 77 |
| Habenula Nucleus_right | 21 |
| Hippocampus_left | 20 |
| Hippocampus_right | 2 |
| Thalamus_left | 2 |
| Cluster12 | 49 |
| Thalamus_right | 8 |
| Cluster13 | 182 |
| DG_right | 6 |
| Hippocampus_right | 13 |
| Subiculum_right | 83 |
| Cluster14 | 87 |
| DG_right | 33 |
| Habenula Nucleus_right | 7 |
| Thalamus_right | 4 |
| Cluster15 | 65 |
| RSC_right | 17 |
| Visual Cortex_right | 49 |
| Cluster16 | 27 |
| Temporal Cortex_right | 4 |
| Visual Cortex_right | 27 |
| Cluster17 | 23 |
| Auditory Cortex_left | 23 |

**Supplementary Table 4. List of the 106 brain subregions in the pain-related network**

| Node | Region |
| --- | --- |
| AId1 L | AI |
| AId1 R | AI |
| AId2/3 L | AI |
| AId2/3 R | AI |
| AId5 L | AI |
| AId5 R | AI |
| AId6a L | AI |
| AId6a R | AI |
| AId6b L | AI |
| AId6b R | AI |
| AIp1 L | AI |
| AIp1 R | AI |
| AIp2/3 L | AI |
| AIp2/3 R | AI |
| AIp5 L | AI |
| AIp5 R | AI |
| AIp6a L | AI |
| AIp6a R | AI |
| AIp6b L | AI |
| AIp6b R | AI |
| AIv1 L | AI |
| AIv1 R | AI |
| AIv2/3 L | AI |
| AIv2/3 R | AI |
| AIv5 L | AI |
| AIv5 R | AI |
| AIv6a L | AI |
| AIv6a R | AI |
| AIv6b L | AI |
| AIv6b R | AI |
| BLAa L | Amygdala |
| BLAa R | Amygdala |
| BLAp L | Amygdala |
| BLAp R | Amygdala |
| BLAv L | Amygdala |
| BLAv R | Amygdala |
| BMAa L | Amygdala |
| BMAa R | Amygdala |
| BMAp L | Amygdala |
| BMAp R | Amygdala |
| CeA-ca L | Amygdala |
| CeA-ca R | Amygdala |
| CeA-la L | Amygdala |
| CeA-la R | Amygdala |
| CeA-me L | Amygdala |
| CeA-me R | Amygdala |
| ACAd1 L | ACC |
| ACAd1 R | ACC |
| ACAd2/3 L | ACC |
| ACAd2/3 R | ACC |
| ACAd5 L | ACC |
| ACAd5 R | ACC |
| ACAd6 L | ACC |
| ACAd6 R | ACC |
| ACAv1 L | ACC |
| ACAv1 R | ACC |
| ACAv2/3 L | ACC |
| ACAv2/3 R | ACC |
| ACAv5 L | ACC |
| ACAv5 R | ACC |
| ACAv6a L | ACC |
| ACAv6a R | ACC |
| ACAv6b L | ACC |
| ACAv6b R | ACC |
| CA1 L | Hippocampus |
| CA1 R | Hippocampus |
| CA2 L | Hippocampus |
| CA2 R | Hippocampus |
| CA3 L | Hippocampus |
| CA3 R | Hippocampus |
| DG-mo L | Hippocampus |
| DG-mo R | Hippocampus |
| DG-po L | Hippocampus |
| DG-po R | Hippocampus |
| DG-sg L | Hippocampus |
| DG-sg R | Hippocampus |
| LC L | LC |
| LC R | LC |
| MD L | Medial thalamus |
| MD R | Medial thalamus |
| SubMD L | Medial thalamus |
| SubMD R | Medial thalamus |
| CeMD L | Medial thalamus |
| CeMD R | Medial thalamus |
| ClMD L | Medial thalamus |
| ClMD R | Medial thalamus |
| PL1 L | mPFC |
| PL1 R | mPFC |
| PL2/3 L | mPFC |
| PL2/3 R | mPFC |
| PL4/5 L | mPFC |
| PL4/5 R | mPFC |
| PL6a L | mPFC |
| PL6a R | mPFC |
| IL1 L | mPFC |
| IL1 R | mPFC |
| IL2/3 L | mPFC |
| IL2/3 R | mPFC |
| IL4/5 L | mPFC |
| IL4/5 R | mPFC |
| IL6a L | mPFC |
| IL6a R | mPFC |
| PAG L | PAG |
| PAG R | PAG |
| SPAG L | PAG |
| SPAG R | PAG |
